# Supplementary material for: Gene signature discovery and systematic validation across diverse clinical cohorts for TB prognosis and response to treatment
Source: PLoS Comput Biol. 2023 Jul 20;19(7):e1010770. doi: 10.1371/journal.pcbi.1010770 (PMC10393163; doi:10.1371/journal.pcbi.1010770)
Supplement: S2 Table — Results from gene enrichment analysis on 45 candidate genes using REACTOME gene sets. (PDF) [file pcbi.1010770.s002.pdf]

Term	Overlap	P-value	Adjusted P-value	Genes
Interferon Signaling	9/196	4.53E-10	1.14E-07	GBP5, RSAD2, STAT1, JAK2, GBP1, FCGR1B, IFIT3, GBP4, IFIT2
Immune System	18/1547	2.03E-09	2.55E-07	C1QB, CD274, GBP5, DUSP3, RSAD2, STAT1, LY96, MAPK14, IFIT3, IFIT2, AIM2, CD19, FBXO6, JAK2, TLR5, GBP1, FCGR1B, GBP4
Interferon gamma signaling	6/93	6.04E-08	5.08E-06	GBP5, STAT1, JAK2, GBP1, FCGR1B, GBP4
Cytokine Signaling in Immune system	9/620	8.15E-06	5.13E-04	GBP5, RSAD2, STAT1, JAK2, GBP1, FCGR1B, IFIT3, GBP4, IFIT2
Interferon alpha/beta signaling	4/68	1.64E-05	8.26E-04	RSAD2, STAT1, IFIT3, IFIT2
Interleukin-6 signaling	2/11	2.69E-04	0.009873	STAT1, JAK2
Toll-Like Receptors Cascades	4/140	2.74E-04	0.009873	DUSP3, LY96, MAPK14, TLR5

S2 Table. Pathway enrichment analysis of the common gene signatures. Results from gene enrichment analysis on 45 candidate genes using REACTOME gene sets.
