# Supplementary material for: Gene signature discovery and systematic validation across diverse clinical cohorts for TB prognosis and response to treatment
Source: PLoS Comput Biol. 2023 Jul 20;19(7):e1010770. doi: 10.1371/journal.pcbi.1010770 (PMC10393163; doi:10.1371/journal.pcbi.1010770)
Supplement: S3 Table — 5-fold nested cross-validation performance metrics among seven selected machine-learning approaches using the pooled discovery datasets (27 cohorts, datapoints n = 2914). Outer CV AUROC presented graphically in S6 Fig. (PDF) [file pcbi.1010770.s003.pdf]

ML type	Cross Validation fold	Inner CV mean squared error	Outer CV mean squared error	Outer CV r2	Outer CV AUROC	# features
Support vector machine	Fold 1	0.12535	0.134806736	0.422078385	0.888469	35
	Fold 2	0.12223	0.16202878	0.271557799	0.835513	37
	Fold 3	0.1232	0.145485714	0.368675246	0.882899	36
	Fold 4	0.12161	0.121276732	0.449320213	0.896788	42
	Fold 5	0.12477	0.139605354	0.353392267	0.87495	33
Random forest	Fold 1	0.10824	0.115712122	0.47189422	0.900731	35
	Fold 2	0.11112	0.104998793	0.531890915	0.923051	37
	Fold 3	0.10725	0.102516937	0.529520189	0.921499	36
	Fold 4	0.10975	0.105128641	0.519461024	0.920225	42
	Fold 5	0.11133	0.107287322	0.505775306	0.914664	33
Elastic net	Fold 1	0.16553	0.177626051	0.177432507	0.811288	35
	Fold 2	0.16847	0.167222478	0.223634104	0.847927	37
	Fold 3	0.16646	0.173145045	0.197150056	0.826173	36
	Fold 4	0.16898	0.171061369	0.20658821	0.839441	42
	Fold 5	0.16886	0.162171398	0.246372257	0.832584	33
Adaptive boosting	Fold 1	0.13479	0.146447768	0.331186836	0.8472	35
	Fold 2	0.14148	0.136208534	0.389743832	0.872539	37
	Fold 3	0.1333	0.132927767	0.395496792	0.876505	36
	Fold 4	0.13346	0.141352288	0.346929548	0.861884	42
	Fold 5	0.13776	0.135681254	0.385407643	0.870585	33
Partial least squares	Fold 1	0.16383	0.178489261	0.174899137	0.809612	35
	Fold 2	0.1677	0.166340333	0.228971917	0.851073	37
	Fold 3	0.1656	0.173703028	0.196260158	0.821547	36
	Fold 4	0.16757	0.169972661	0.21199362	0.836877	42
	Fold 5	0.16837	0.163176985	0.241745793	0.822385	33
Multilayer perceptron	Fold 1	0.11068	0.120884813	0.442106526	0.895058	35
	Fold 2	0.11319	0.113109607	0.492675002	0.881656	37
	Fold 3	0.11321	0.112396706	0.484707383	0.896247	36
	Fold 4	0.11772	0.10990995	0.49043002	0.903789	42
	Fold 5	0.11553	0.116177792	0.485655834	0.907721	33
Extreme gradient boosting	Fold 1	0.10796	0.141314945	0.349845073	0.854219	35
	Fold 2	0.11344	0.119385948	0.455040281	0.899911	37
	Fold 3	0.11076	0.140167115	0.389811924	0.874422	36
	Fold 4	0.11200	0.122377948	0.444486009	0.893244	42
	Fold 5	0.11381	0.132638969	0.423787823	0.891314	33

S3 Table. 5-fold nested cross-validation performance metrics among seven selected machine-learning approaches using the pooled discovery datasets (27 cohorts, datapoints $n = 2914$). Outer CV AUROC presented graphically in **S6 Fig**.
