# Supplementary material for: Gene signature discovery and systematic validation across diverse clinical cohorts for TB prognosis and response to treatment
Source: PLoS Comput Biol. 2023 Jul 20;19(7):e1010770. doi: 10.1371/journal.pcbi.1010770 (PMC10393163; doi:10.1371/journal.pcbi.1010770)
Supplement: S4 Table — The parameters of the final models trained by the pooled discovery dataset (27 cohorts). (PDF) [file pcbi.1010770.s004.pdf]

Model	parameters	# features	Features (paired genes)	# genes selected in features	Genes
Full model	RandomForestRegressor (bootstrap=False, max_depth=30, max_features='sqrt', n_estimators=700, random_state=1)	41	SPOCK2_DUSP3, SPOCK2_STAT1, CD19_SERPING1, CD19_LAP3, FBXO6_C1QB, C1QB_SLC6A12, C1QB_RTP4, CD19_DUSP3, GK_LRRK2, SPOCK2_IFIT2, PSTPIP2_SERPING1, ZNF438_LHFPL2, SPOCK2_CD5, FBXO6_FCGR1B, CD274_MAPK14, AIM2_SERPING1, BATF2_ANKRD22, LRRK2_LMNB1, CD274_APOL6, HP_C1QB, PSTPIP2_LHFPL2, SAMD9L_LAP3, CD274_JAK2, NELL2_CD5, BATF2_C1QB, CASP5_FCGR1B, GBP5_GBP4, CD274_ZNF438, CD274_P2RY14, TIMM10_FBXO6, DUSP3_LY96, FBXO6_GBP1, TLR5_ZNF438, ADM_IFIT2, RSAD2_IFIT3, RSAD2_GBP5, RSAD2_FCGR1B, LRRK2_KCNJ15, GBP4_LAP3, FBXO6_VAMP5, ZNF438_KCNJ15	42	ADM, AIM2, ANKRD22, APOL6, BATF2, C1QB, CASP5, CD19, CD274, CD5, DUSP3, GBP1, GBP4, GBP5, FBXO6, FCGR1B, GK, HP, IFIT2, IFIT3, JAK2, KCNJ15, LAP3, LHFPL2, LMNB1, LRRK2, LY96, MAPK14, NELL2, P2RY14, PSTPIP2, RSAD2, RTP4, SAMD9L, SERPING1, SLC6A12, SPOCK2, STAT1, TIMM10, TLR5, VAMP5, ZNF438
Reduced model	RandomForestRegressor (bootstrap=False, max_depth=90, max_features='sqrt', n_estimators=1400, random_state=1)	12	FBXO6_VAMP5, LRRK2_LMNB1, BATF2_ANKRD22, SPOCK2_DUSP3, CD274_NELL2, GBP5_GBP4, IFIT2_ADM, ZNF438_FCGR1B, NELL2_CD5, CD274_APOL6, SPOCK2_CD5, IFIT2_SPOCK2	18	ADM, ANKRD22, APOL6, BATF2, CD274, CD5, DUSP3, GBP4, GBP5, FBXO6, FCGR1B, LMNB1, LRRK2, IFIT2, NELL2, SPOCK2, VAMP5, ZNF438

S4 Table. The parameters of the final models trained by the pooled discovery dataset (27 cohorts).
