# Supplementary material for: Gene signature discovery and systematic validation across diverse clinical cohorts for TB prognosis and response to treatment
Source: PLoS Comput Biol. 2023 Jul 20;19(7):e1010770. doi: 10.1371/journal.pcbi.1010770 (PMC10393163; doi:10.1371/journal.pcbi.1010770)
Supplement: S5 Table — Prognostic performance of the new models developed in this report and published previously for incipient TB, stratified by time interval to disease, using cut-offs identified by the maximal Youden Index based on the best tradeoff between sensitivity and specificity from each ROC. Positive and negative predictive values (PPVs/NPVs) were calculated when assuming 2% pre-test probability. The performance metrics are presented with 95% confidence interval. (PDF) [file pcbi.1010770.s005.pdf]

	Time interval	Sensitivity	Specificity	PPV	NPV
Full model	< 3m to disease	0.873 (0.815 - 0.931)	0.917 (0.899 - 0.935)	0.177 (0.145 - 0.209)	0.997 (0.996 - 0.998)
	< 6m to disease	0.821 (0.764 - 0.878)	0.838 (0.813 - 0.862)	0.093 (0.081 - 0.106)	0.996 (0.995 - 0.997)
	< 12m to disease	0.786 (0.734 - 0.839)	0.803 (0.777 - 0.830)	0.075 (0.066 - 0.085)	0.995 (0.994 - 0.995)
	< 18m to disease	0.773 (0.723 - 0.823)	0.783 (0.756 - 0.810)	0.068 (0.060 - 0.076)	0.994 (0.993 - 0.995)
	< 24m to disease	0.747 (0.697 - 0.796)	0.783 (0.756 - 0.810)	0.066 (0.058 - 0.073)	0.993 (0.993 - 0.994)
	< 30m to disease	0.742 (0.693 - 0.792)	0.783 (0.756 - 0.810)	0.065 (0.058 - 0.073)	0.993 (0.993 - 0.994)
Reduced model	< 3m to disease	0.833 (0.768 - 0.898)	0.916 (0.898 - 0.934)	0.168 (0.138 - 0.199)	0.996 (0.995 - 0.997)
	< 6m to disease	0.740 (0.675 - 0.805)	0.869 (0.847 - 0.892)	0.104 (0.088 - 0.119)	0.994 (0.993 - 0.995)
	< 12m to disease	0.748 (0.692 - 0.804)	0.810 (0.784 - 0.836)	0.074 (0.065 - 0.084)	0.994 (0.993 - 0.995)
	< 18m to disease	0.784 (0.735 - 0.834)	0.749 (0.720 - 0.778)	0.060 (0.053 - 0.066)	0.994 (0.993 - 0.995)
	< 24m to disease	0.771 (0.722 - 0.819)	0.749 (0.720 - 0.778)	0.059 (0.053 - 0.065)	0.994 (0.993 - 0.995)
	< 30m to disease	0.773 (0.725 - 0.820)	0.749 (0.720 - 0.778)	0.059 (0.053 - 0.065)	0.994 (0.993 - 0.995)
Sweeney 3	< 3m to disease	0.714 (0.635 - 0.793)	0.464 (0.431 - 0.497)	0.026 (0.025 - 0.028)	0.988 (0.986 - 0.989)
	< 6m to disease	0.751 (0.687 - 0.816)	0.401 (0.369 - 0.434)	0.025 (0.023 - 0.026)	0.988 (0.986 - 0.989)
	< 12m to disease	0.752 (0.697 - 0.807)	0.401 (0.369 - 0.434)	0.025 (0.024 - 0.026)	0.988 (0.986 - 0.989)
	< 18m to disease	0.725 (0.672 - 0.778)	0.401 (0.369 - 0.434)	0.024 (0.023 - 0.026)	0.986 (0.985 - 0.987)
	< 24m to disease	0.705 (0.653 - 0.758)	0.401 (0.369 - 0.434)	0.023 (0.022 - 0.025)	0.985 (0.984 - 0.986)
	< 30m to disease	0.709 (0.658 - 0.761)	0.401 (0.369 - 0.434)	0.024 (0.022 - 0.025)	0.985 (0.984 - 0.987)
RISK 6	< 3m to disease	0.857 (0.796 - 0.918)	0.865 (0.842 - 0.887)	0.115 (0.098 - 0.131)	0.997 (0.996 - 0.998)
	< 6m to disease	0.798 (0.738 - 0.858)	0.836 (0.812 - 0.861)	0.090 (0.078 - 0.103)	0.995 (0.994 - 0.996)
	< 12m to disease	0.752 (0.697 - 0.807)	0.836 (0.812 - 0.861)	0.086 (0.074 - 0.097)	0.994 (0.993 - 0.995)
	< 18m to disease	0.714 (0.660 - 0.768)	0.836 (0.812 - 0.861)	0.082 (0.071 - 0.093)	0.993 (0.992 - 0.994)
	< 24m to disease	0.740 (0.689 - 0.790)	0.761 (0.733 - 0.790)	0.059 (0.053 - 0.066)	0.993 (0.992 - 0.994)
	< 30m to disease	0.732 (0.682 - 0.783)	0.761 (0.733 - 0.790)	0.059 (0.052 - 0.065)	0.993 (0.992 - 0.994)
BATF2	< 3m to disease	0.738 (0.661 - 0.815)	0.594 (0.562 - 0.627)	0.036 (0.033 - 0.039)	0.991 (0.990 - 0.992)
	< 6m to disease	0.717 (0.650 - 0.784)	0.555 (0.522 - 0.587)	0.032 (0.029 - 0.034)	0.990 (0.989 - 0.991)
	< 12m to disease	0.662 (0.602 - 0.723)	0.549 (0.516 - 0.582)	0.029 (0.027 - 0.031)	0.988 (0.987 - 0.989)
	< 18m to disease	0.721 (0.668 - 0.775)	0.476 (0.443 - 0.509)	0.027 (0.026 - 0.029)	0.988 (0.987 - 0.989)
	< 24m to disease	0.709 (0.657 - 0.761)	0.472 (0.439 - 0.505)	0.027 (0.025 - 0.028)	0.988 (0.987 - 0.989)
	< 30m to disease	0.702 (0.651 - 0.754)	0.472 (0.439 - 0.505)	0.026 (0.025 - 0.028)	0.987 (0.986 - 0.988)
Suliman 4	< 3m to disease	0.841 (0.777 - 0.905)	0.831 (0.806 - 0.855)	0.092 (0.080 - 0.104)	0.996 (0.995 - 0.997)
	< 6m to disease	0.746 (0.681 - 0.811)	0.831 (0.806 - 0.855)	0.082 (0.071 - 0.094)	0.994 (0.993 - 0.995)
	< 12m to disease	0.667 (0.606 - 0.727)	0.831 (0.806 - 0.855)	0.074 (0.064 - 0.084)	0.992 (0.991 - 0.993)
	< 18m to disease	0.617 (0.559 - 0.675)	0.831 (0.806 - 0.855)	0.069 (0.060 - 0.079)	0.991 (0.990 - 0.992)
	< 24m to disease	0.716 (0.664 - 0.767)	0.670 (0.639 - 0.702)	0.042 (0.039 - 0.046)	0.991 (0.991 - 0.99
	< 30m to disease	0.712 (0.661 - 0.764)	0.670 (0.639 - 0.702)	0.042 (0.038 - 0.046)	0.991 (0.991 - 0.992)

S5 Table. Prognostic performance of the new models developed in this report and published previously for incipient TB, stratified by time interval to disease, using cut-offs identified by the maximal Youden Index based on the best tradeoff between sensitivity and specificity from each ROC. Positive and negative predictive values (PPVs/NPVs) were calculated when assuming 2% pre-test probability. The performance metrics are presented with 95% confidence interval.
