# Supplementary material for: Gene signature discovery and systematic validation across diverse clinical cohorts for TB prognosis and response to treatment
Source: PLoS Comput Biol. 2023 Jul 20;19(7):e1010770. doi: 10.1371/journal.pcbi.1010770 (PMC10393163; doi:10.1371/journal.pcbi.1010770)
Supplement: S6 Table — Prognostic performance of the models developed in this report and published previously for incipient TB, stratified by time interval to disease, using the predetermined cutoffs defined by 2 standard deviations (SDs) above the mean of the control group (latent TB infection without progression during follow-up). Positive and negative predictive values (PPVs/NPVs) were calculated when assuming 2% pre-test probability. The performance metrics presented with 95% confidence interval. (PDF) [file pcbi.1010770.s006.pdf]

	Time interval	Sensitivity	Specificity	PPV	NPV
Full model	< 3m to disease	0.667 (0.584 - 0.749)	0.965 (0.953 - 0.977)	0.279 (0.209 - 0.348)	0.993 (0.991 - 0.995)
	< 6m to disease	0.561 (0.487 - 0.635)	0.965 (0.953 - 0.977)	0.245 (0.181 - 0.309)	0.991 (0.989 - 0.992)
	< 12m to disease	0.487 (0.423 - 0.551)	0.965 (0.953 - 0.977)	0.220 (0.161 - 0.279)	0.989 (0.988 - 0.990)
	< 18m to disease	0.446 (0.387 - 0.506)	0.965 (0.953 - 0.977)	0.205 (0.149 - 0.262)	0.988 (0.987 - 0.990)
	< 24m to disease	0.418 (0.361 - 0.474)	0.965 (0.953 - 0.977)	0.195 (0.141 - 0.249)	0.988 (0.987 - 0.989)
	< 30m to disease	0.411 (0.356 - 0.467)	0.965 (0.953 - 0.977)	0.192 (0.139 - 0.246)	0.988 (0.987 - 0.989)
Reduced model	< 3m to disease	0.690 (0.610 - 0.771)	0.967 (0.955 - 0.979)	0.300 (0.224 - 0.375)	0.994 (0.992 - 0.995)
	< 6m to disease	0.566 (0.493 - 0.640)	0.967 (0.955 - 0.979)	0.260 (0.191 - 0.329)	0.991 (0.990 - 0.992)
	< 12m to disease	0.483 (0.419 - 0.547)	0.967 (0.955 - 0.979)	0.230 (0.167 - 0.294)	0.989 (0.988 - 0.990)
	< 18m to disease	0.439 (0.379 - 0.498)	0.967 (0.955 - 0.979)	0.214 (0.153 - 0.274)	0.988 (0.987 - 0.989)
	< 24m to disease	0.418 (0.361 - 0.474)	0.967 (0.955 - 0.979)	0.206 (0.147 - 0.264)	0.988 (0.987 - 0.989)
	< 30m to disease	0.411 (0.356 - 0.467)	0.967 (0.955 - 0.979)	0.203 (0.145 - 0.261)	0.988 (0.987 - 0.989)
Sweeney 3	< 3m to disease	0.048 (0.010 - 0.085)	0.990 (0.983 - 0.996)	0.087 (0.035 - 0.138)	0.981 (0.980 - 0.981)
	< 6m to disease	0.046 (0.015 - 0.078)	0.990 (0.983 - 0.996)	0.084 (0.034 - 0.135)	0.981 (0.980 - 0.981)
	< 12m to disease	0.056 (0.026 - 0.085)	0.990 (0.983 - 0.996)	0.100 (0.041 - 0.158)	0.981 (0.980 - 0.981)
	< 18m to disease	0.048 (0.023 - 0.074)	0.990 (0.983 - 0.996)	0.088 (0.036 - 0.140)	0.981 (0.980 - 0.981)
	< 24m to disease	0.045 (0.021 - 0.068)	0.990 (0.983 - 0.996)	0.082 (0.033 - 0.130)	0.981 (0.980 - 0.981)
	< 30m to disease	0.043 (0.020 - 0.067)	0.990 (0.983 - 0.996)	0.080 (0.032 - 0.128)	0.981 (0.980 - 0.981)
RISK 6	< 3m to disease	0.643 (0.559 - 0.727)	0.965 (0.953 - 0.977)	0.271 (0.203 - 0.340)	0.993 (0.991 - 0.994)
	< 6m to disease	0.549 (0.475 - 0.623)	0.965 (0.953 - 0.977)	0.241 (0.178 - 0.305)	0.991 (0.989 - 0.992)
	< 12m to disease	0.474 (0.410 - 0.538)	0.965 (0.953 - 0.977)	0.216 (0.157 - 0.274)	0.989 (0.988 - 0.990)
	< 18m to disease	0.428 (0.368 - 0.487)	0.965 (0.953 - 0.977)	0.199 (0.143 - 0.254)	0.988 (0.987 - 0.989)
	< 24m to disease	0.408 (0.351 - 0.464)	0.965 (0.953 - 0.977)	0.191 (0.138 - 0.244)	0.988 (0.987 - 0.989)
	< 30m to disease	0.401 (0.346 - 0.457)	0.965 (0.953 - 0.977)	0.189 (0.136 - 0.242)	0.987 (0.986 - 0.989)
BATF2	< 3m to disease	0.016 (0.000 - 0.038)	0.967 (0.955 - 0.979)	0.010 (0.006 - 0.013)	0.980 (0.979 - 0.980)
	< 6m to disease	0.023 (0.001 - 0.046)	0.967 (0.955 - 0.979)	0.014 (0.009 - 0.019)	0.980 (0.979 - 0.980)
	< 12m to disease	0.030 (0.008 - 0.052)	0.967 (0.955 - 0.979)	0.018 (0.012 - 0.025)	0.980 (0.979 - 0.980)
	< 18m to disease	0.026 (0.007 - 0.045)	0.967 (0.955 - 0.979)	0.016 (0.010 - 0.021)	0.980 (0.979 - 0.980)
	< 24m to disease	0.024 (0.006 - 0.042)	0.967 (0.955 - 0.979)	0.015 (0.009 - 0.020)	0.980 (0.979 - 0.980)
	< 30m to disease	0.023 (0.006 - 0.041)	0.967 (0.955 - 0.979)	0.014 (0.009 - 0.019)	0.980 (0.979 - 0.980)
Suliman 4	< 3m to disease	0.563 (0.477 - 0.650)	0.966 (0.954 - 0.978)	0.252 (0.186 - 0.319)	0.991 (0.989 - 0.993)
	< 6m to disease	0.491 (0.417 - 0.566)	0.966 (0.954 - 0.978)	0.227 (0.166 - 0.289)	0.989 (0.988 - 0.991)
	< 12m to disease	0.419 (0.356 - 0.482)	0.966 (0.954 - 0.978)	0.200 (0.144 - 0.257)	0.988 (0.987 - 0.989)
	< 18m to disease	0.379 (0.321 - 0.437)	0.966 (0.954 - 0.978)	0.185 (0.132 - 0.238)	0.987 (0.986 - 0.988)
	< 24m to disease	0.360 (0.305 - 0.415)	0.966 (0.954 - 0.978)	0.177 (0.126 - 0.228)	0.987 (0.986 - 0.988)
	< 30m to disease	0.355 (0.300 - 0.409)	0.966 (0.954 - 0.978)	0.175 (0.124 - 0.226)	0.987 (0.986 - 0.988)

S6 Table. Prognostic performance of the models developed in this report and published previously for incipient TB, stratified by time interval to disease, using the predetermined cutoffs defined by 2 standard deviations (SDs) above the mean of the control group (latent TB infection without progression during follow-up). Positive and negative predictive values (PPVs/NPVs) were calculated when assuming 2% pre-test probability. The performance metrics presented with 95% confidence interval.
