# Supplementary material for: Gene signature discovery and systematic validation across diverse clinical cohorts for TB prognosis and response to treatment
Source: PLoS Comput Biol. 2023 Jul 20;19(7):e1010770. doi: 10.1371/journal.pcbi.1010770 (PMC10393163; doi:10.1371/journal.pcbi.1010770)
Supplement: S1 Fig — (A-D) Null distribution of edge weights from permuting log2(Fold Change) values across cohorts and calculating dot products compared against the actual distribution of edge weights from the matrix of log2(Fold Change) values for a given disease comparison (Methods). Edge weights between -2 and 2 are likely to arise by chance and are discarded during network construction. (PDF) [file pcbi.1010770.s007.pdf]

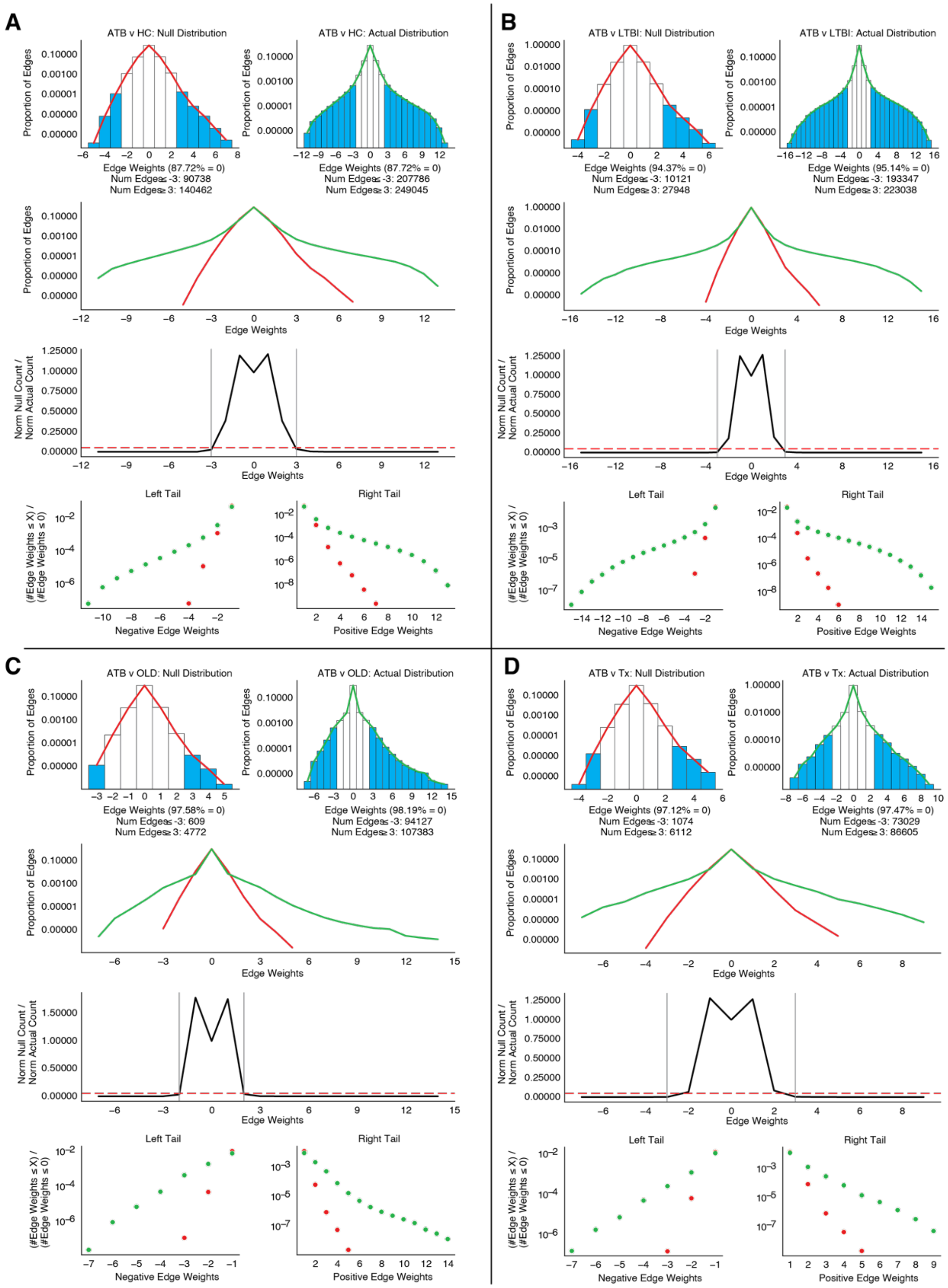

S1 Fig. Network building parameter selection. (**A-D**) Null distribution of edge weights from permuting log2(Fold Change) values across cohorts and calculating dot products compared against the actual distribution of edge weights from the matrix of log2(Fold Change) values for a given disease comparison (Methods). Edge weights between -2 and 2 are likely to arise by chance and are discarded during network construction.
