# Supplementary material for: Gene signature discovery and systematic validation across diverse clinical cohorts for TB prognosis and response to treatment
Source: PLoS Comput Biol. 2023 Jul 20;19(7):e1010770. doi: 10.1371/journal.pcbi.1010770 (PMC10393163; doi:10.1371/journal.pcbi.1010770)
Supplement: S2 Fig — Distribution of degree, weighted degree, and eigenvector centrality for the four different networks constructed from (A) ATB vs. HC, (B) ATB vs. LTBI, (C) ATB vs. OLD, and (D) ATB vs. Tx disease comparisons. (PDF) [file pcbi.1010770.s008.pdf]

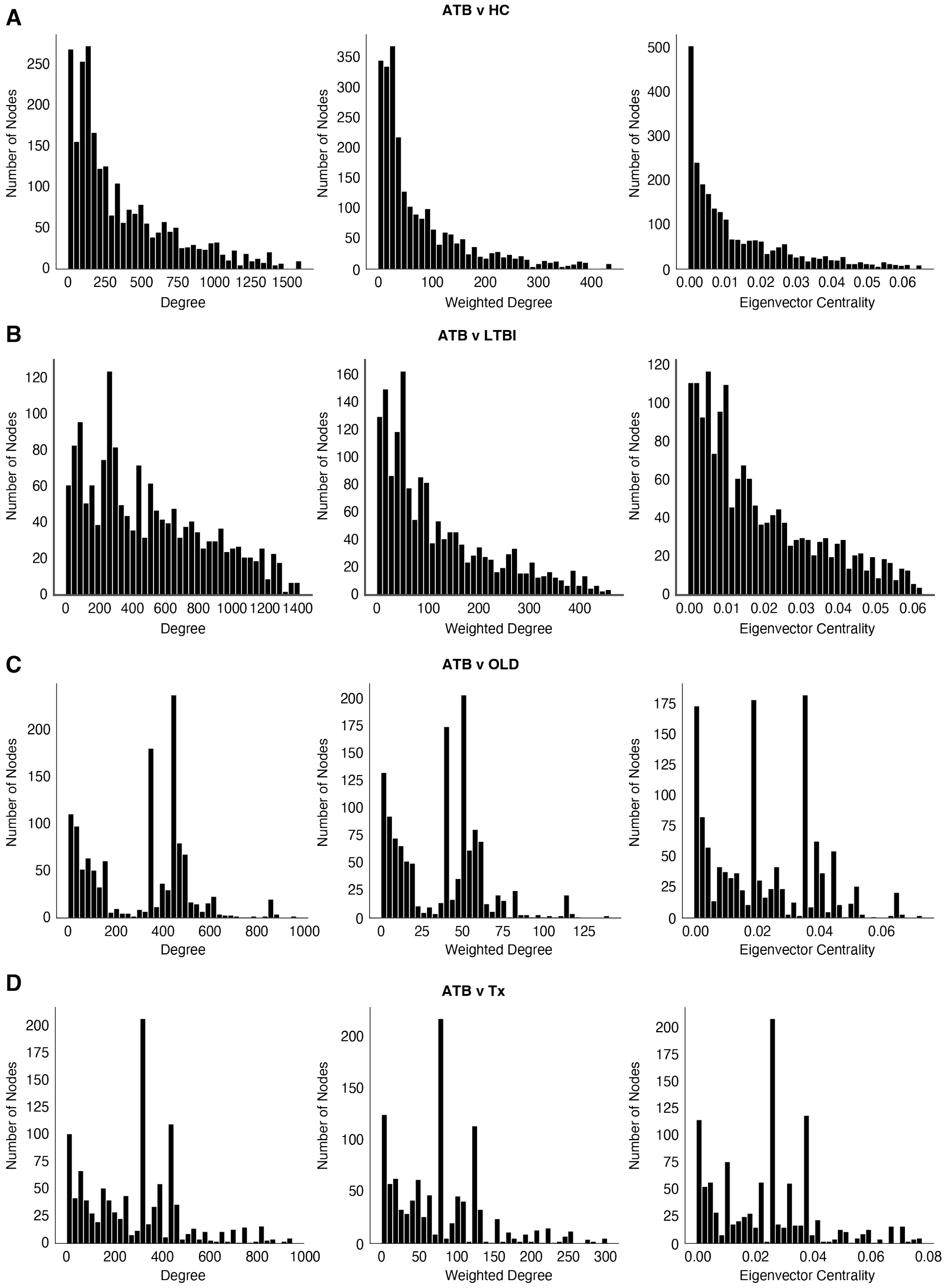

S2 Fig. Network degree distribution. Distribution of degree, weighted degree, and eigenvector centrality for the four different networks constructed from (**A**) ATB vs. HC, (**B**) ATB vs. LTBI, (**C**) ATB vs. OLD, and (**D**) ATB vs. Tx disease comparisons.
