# Supplementary material for: Gene signature discovery and systematic validation across diverse clinical cohorts for TB prognosis and response to treatment
Source: PLoS Comput Biol. 2023 Jul 20;19(7):e1010770. doi: 10.1371/journal.pcbi.1010770 (PMC10393163; doi:10.1371/journal.pcbi.1010770)
Supplement: S3 Fig — Pearson r correlation coefficient calculated between mean log2(Fold Change) between each pair of comparisons (ATB v HC, ATB v LTBI, ATB v OLD, ATB v Tx) for the 45 gene set. Correlations were computed by first averaging the values column-wise (across datasets) for the data in each heatmap Fig 2L–2O to calculate at vector of length 45 for each comparison, then calculating the Pearson r correlation (as implemented in scipy python package) between each pair of vectors. Correlation values and corresponding p-values are reported in the correlation matrix below. (PDF) [file pcbi.1010770.s009.pdf]

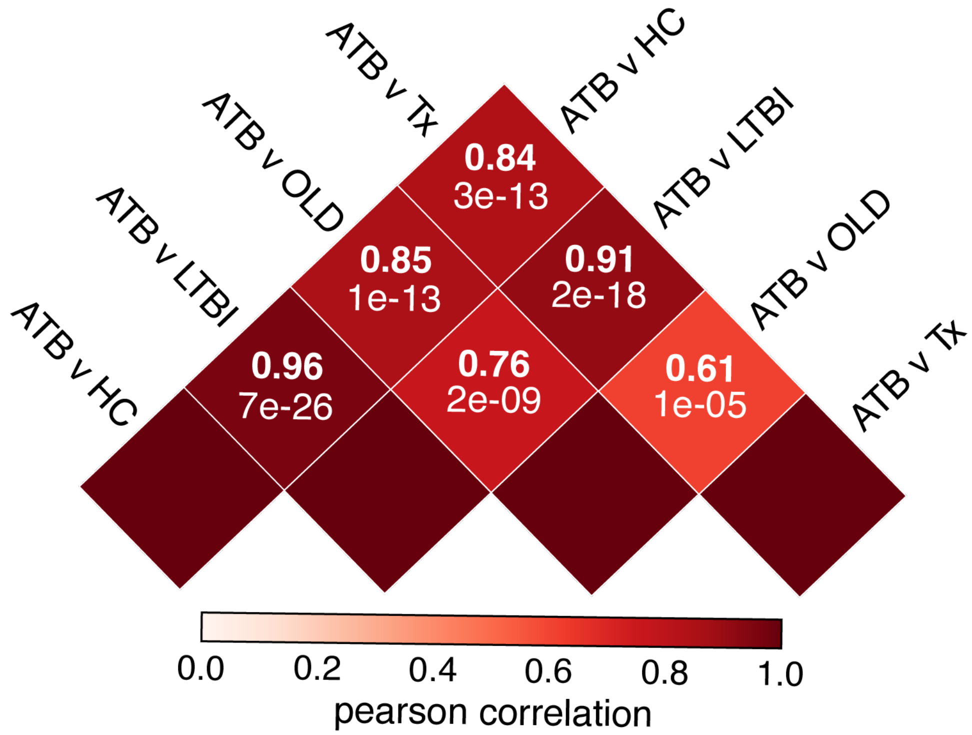

S3 Fig. Pearson r correlation coefficient calculated between mean log2(Fold Change) between each pair of comparisons (ATB v HC, ATB v LTBI, ATB v OLD, ATB v Tx) for the 45 gene set. Correlations were computed by first averaging the values column-wise (across datasets) for the data in each heatmap **Fig 2L-O** to calculate at vector of length *45* for each comparison, then calculating the Pearson r correlation (as implemented in *scipy* python package) between each pair of vectors. Correlation values and corresponding p-values are reported in the correlation matrix below.
