# Supplementary material for: Gene signature discovery and systematic validation across diverse clinical cohorts for TB prognosis and response to treatment
Source: PLoS Comput Biol. 2023 Jul 20;19(7):e1010770. doi: 10.1371/journal.pcbi.1010770 (PMC10393163; doi:10.1371/journal.pcbi.1010770)
Supplement: S4 Fig — Genes included in our 45-candidate gene set (the first column in both panels) as compared to genes in gene sets from 30 previously published gene signatures (2, 43). We exclude the genes (563 out of total 721 genes) only detected in one gene signature and display 155 genes present in at least two gene signatures and 3 additional genes present only in our 45-candidate gene set. The left panel displays genes detected in our candidate gene set while the right panel displays genes detected in at least two gene signatures. The numbers in parentheses indicate the number of studies identifying the gene. (PDF) [file pcbi.1010770.s010.pdf]

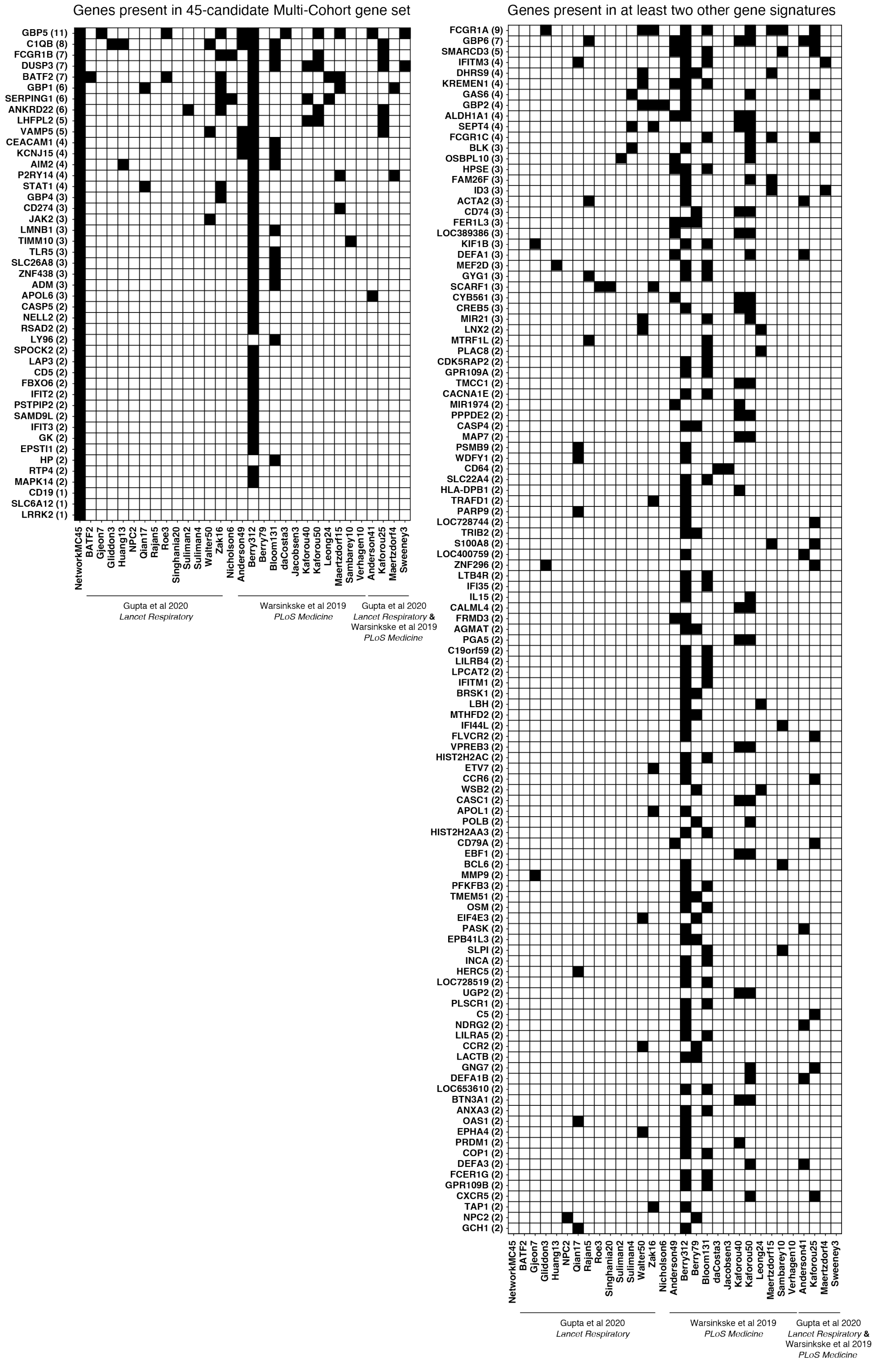

S4 Fig. Heatmap of gene signatures. Genes included in our 45-candidate gene set (the first column in both panels) as compared to genes in gene sets from 30 previously published gene signatures (*2*, *43*). We exclude the genes (563 out of total 721 genes) only detected in one gene signature and display 155 genes present in at least two gene signatures and 3 additional genes present only in our 45-candidate gene set. The left panel displays genes detected in our candidate gene set while the right panel displays genes detected in at least two gene signatures. The numbers in parentheses indicate the number of studies identifying the gene.
