# Supplementary material for: Gene signature discovery and systematic validation across diverse clinical cohorts for TB prognosis and response to treatment
Source: PLoS Comput Biol. 2023 Jul 20;19(7):e1010770. doi: 10.1371/journal.pcbi.1010770 (PMC10393163; doi:10.1371/journal.pcbi.1010770)
Supplement: S5 Fig — Head-to-head comparison of model performance generated from 5-fold nested cross-validation among 7 selected supervised learning models using the pooled discovery datasets (27 cohorts, datapoints n = 2914). (PDF) [file pcbi.1010770.s011.pdf]

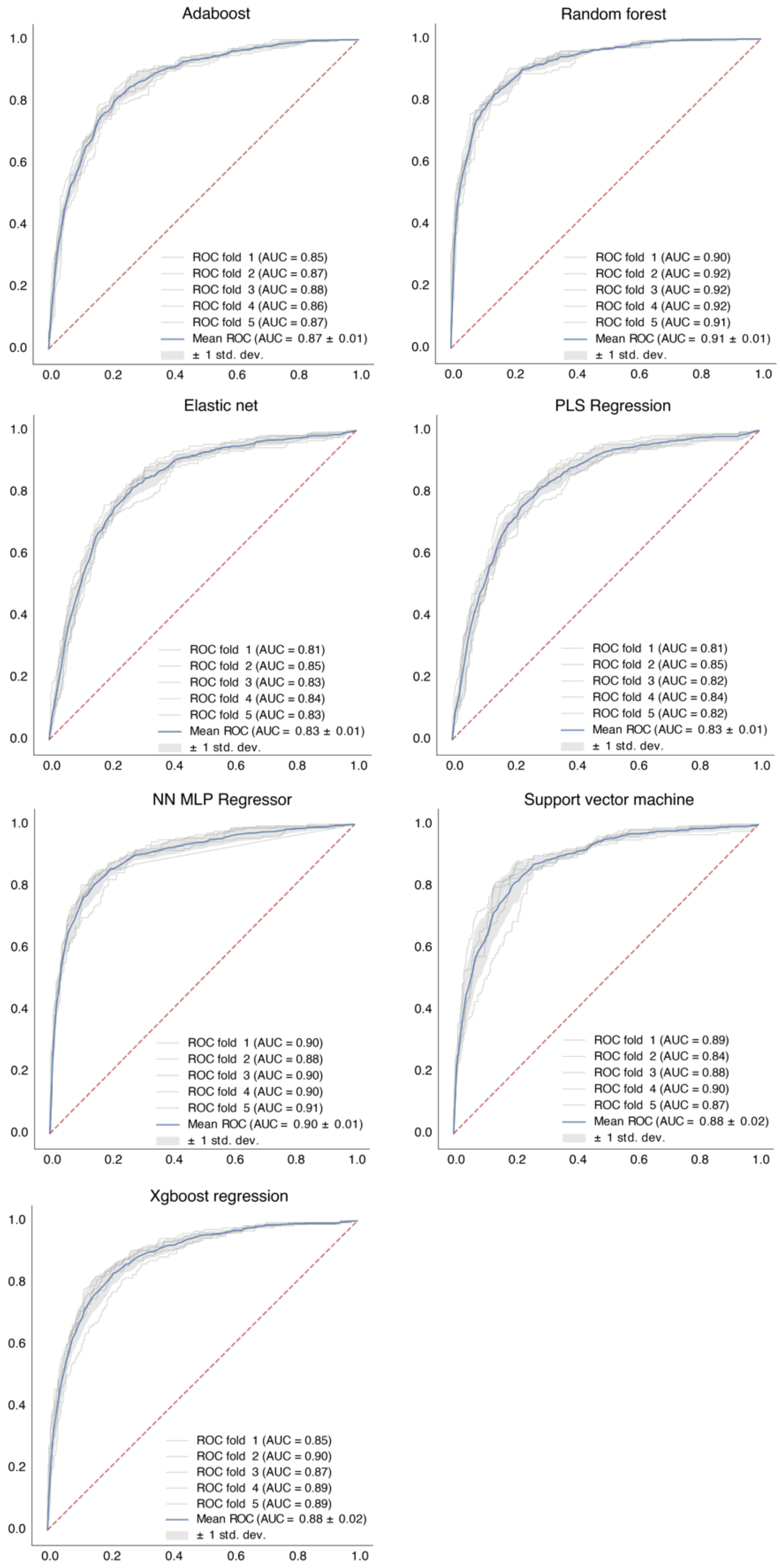

S5 Fig. Head-to-head comparison of model performance generated from 5-fold nested cross-validation among 7 selected supervised learning models using the pooled discovery datasets (27 cohorts, datapoints n = 2914).
