# Supplementary material for: Gene signature discovery and systematic validation across diverse clinical cohorts for TB prognosis and response to treatment
Source: PLoS Comput Biol. 2023 Jul 20;19(7):e1010770. doi: 10.1371/journal.pcbi.1010770 (PMC10393163; doi:10.1371/journal.pcbi.1010770)
Supplement: S6 Fig — (A) The stability path for each feature shown as blue line indicates the probability of a feature being selected from randomly resampling the data as a function of the regularization parameter (λ). Features were ranked by the maximum probability and top 20 features are listed in (B). The top 12 features were selected in our reduced model to minimize the number of features while maintaining CV performance (S7 Fig). (PDF) [file pcbi.1010770.s012.pdf]

A

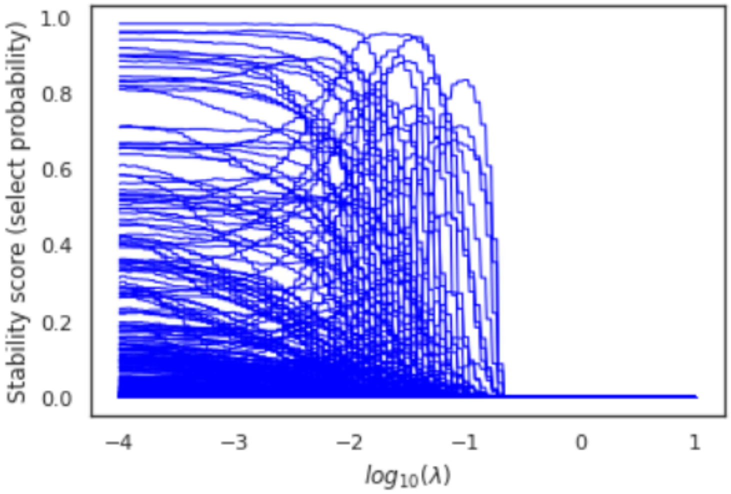

B

**Feature (paired genes) ranking**	**Probability**
VAMP5_FBXO6	0.983
LMNB1_LRRK2	0.965
BATF2_ANKRD22	0.962
SPOCK2_DUSP3	0.957
CD274_NELL2	0.948
GBP5_GBP4	0.941
IFIT2_ADM	0.919
ZNF438_FCGR1B	0.912
NELL2_CD5	0.902
CD274_APOL6	0.9
SPOCK2_CD5	0.897
IFIT2_SPOCK2	0.893
ZNF438_KCNJ15	0.885
GK_LRRK2	0.884
CASP5_FCGR1B	0.869
ZNF438_CD274	0.86
SPOCK2_STAT1	0.844
CD19_C1QB	0.84
NELL2_C1QB	0.835
LY96_DUSP3	0.834

S6 Fig. Feature down-selection by a stability analysis. (**A**) The stability path for each feature shown as blue line indicates the probability of a feature being selected from randomly resampling the data as a function of the regularization parameter (λ). Features were ranked by the maximum probability and top 20 features are listed in (**B**). The top 12 features were selected in our reduced model to minimize the number of features while maintaining CV performance (**S7 Fig**).
