# Supplementary material for: Gene signature discovery and systematic validation across diverse clinical cohorts for TB prognosis and response to treatment
Source: PLoS Comput Biol. 2023 Jul 20;19(7):e1010770. doi: 10.1371/journal.pcbi.1010770 (PMC10393163; doi:10.1371/journal.pcbi.1010770)
Supplement: S7 Fig — The features were ranked by the stability of features selected from randomly resampling the data (S6 Fig). A forward stepwise selection was used to evaluate the model CV performance by adding one feature at a time starting from the top feature. The final feature set was determined when the breakpoint of the AUROC is reached. (PDF) [file pcbi.1010770.s013.pdf]

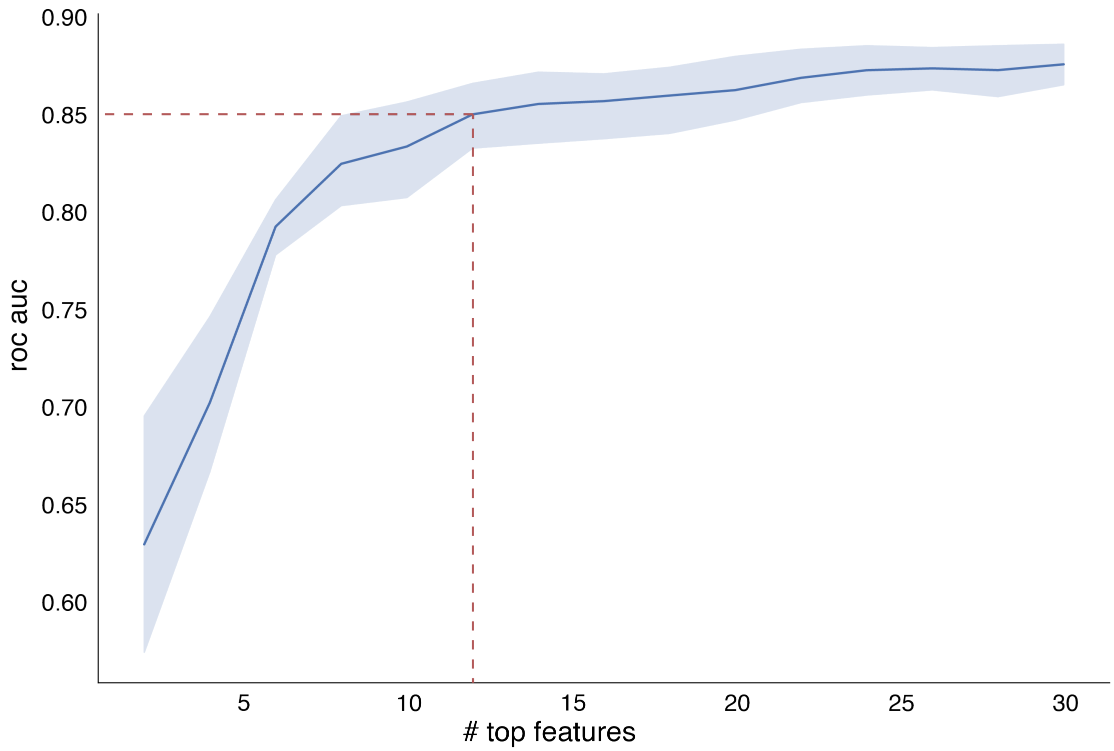

S7 Fig. Feature down-selection of the model based on AUROC with 95% confidence intervals from 5-fold CV. The features were ranked by the stability of features selected from randomly resampling the data (**S6 Fig**). A forward stepwise selection was used to evaluate the model CV performance by adding one feature at a time starting from the top feature. The final feature set was determined when the breakpoint of the AUROC is reached.
