# Supplementary material for: Gene signature discovery and systematic validation across diverse clinical cohorts for TB prognosis and response to treatment
Source: PLoS Comput Biol. 2023 Jul 20;19(7):e1010770. doi: 10.1371/journal.pcbi.1010770 (PMC10393163; doi:10.1371/journal.pcbi.1010770)
Supplement: S8 Fig — The distributions of TB scores generated by the reduced model, stratified by categorical interval to disease, are shown in a violin plot (datapoints n = 1281) (left panel). ROC curves depict prognostic performance for incipient TB, stratified by time intervals to disease (< 3, < 6, <12, <18, < 24, < 30 months) (middle panel) and mutually exclusive time intervals to disease (0–3, 3–6, 6–12, 12–18, 18–24, 24–30 months) (right panel). AUC and 95% confidence intervals for each interval to disease are shown. (PDF) [file pcbi.1010770.s014.pdf]

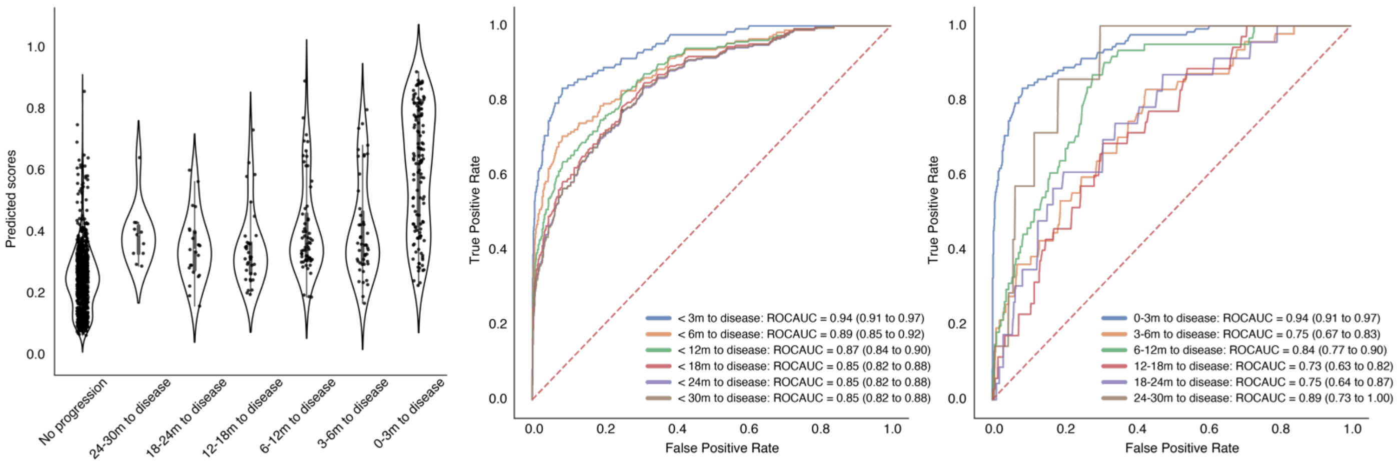

S8 Fig. Prognostic performance of the reduced model for incipient TB using the pooled longitudinal validation datasets. The distributions of TB scores generated by the reduced model, stratified by categorical interval to disease, are shown in a violin plot (datapoints n = 1281) (left panel). ROC curves depict prognostic performance for incipient TB, stratified by time intervals to disease (< 3, < 6, <12, <18, < 24, < 30 months) (middle panel) and mutually exclusive time intervals to disease (0-3, 3-6, 6-12, 12-18, 18-24, 24-30 months) (right panel). AUC and 95% confidence intervals for each interval to disease are shown.
