# Supplementary material for: Gene signature discovery and systematic validation across diverse clinical cohorts for TB prognosis and response to treatment
Source: PLoS Comput Biol. 2023 Jul 20;19(7):e1010770. doi: 10.1371/journal.pcbi.1010770 (PMC10393163; doi:10.1371/journal.pcbi.1010770)
Supplement: S9 Fig — Prognostic performance of the 4 published models (3, 5, 18, 27). (A-D) for incipient TB using the pooled longitudinal validation dataset (6 TB progression studies). The distributions of TB scores, stratified by categorical interval to disease, are shown in a violin plot (datapoints n = 1281) (left panels). ROC curves depict prognostic performance for incipient TB, stratified by time intervals to disease (< 3, < 6, <12, <18, < 24, < 30 months) (middle panel) and mutually exclusive time intervals to disease (0–3, 3–6, 6–12, 12–18, 18–24, 24–30 months) (right panel). AUC and 95% confidence intervals for each interval to disease are shown. (PDF) [file pcbi.1010770.s015.pdf]

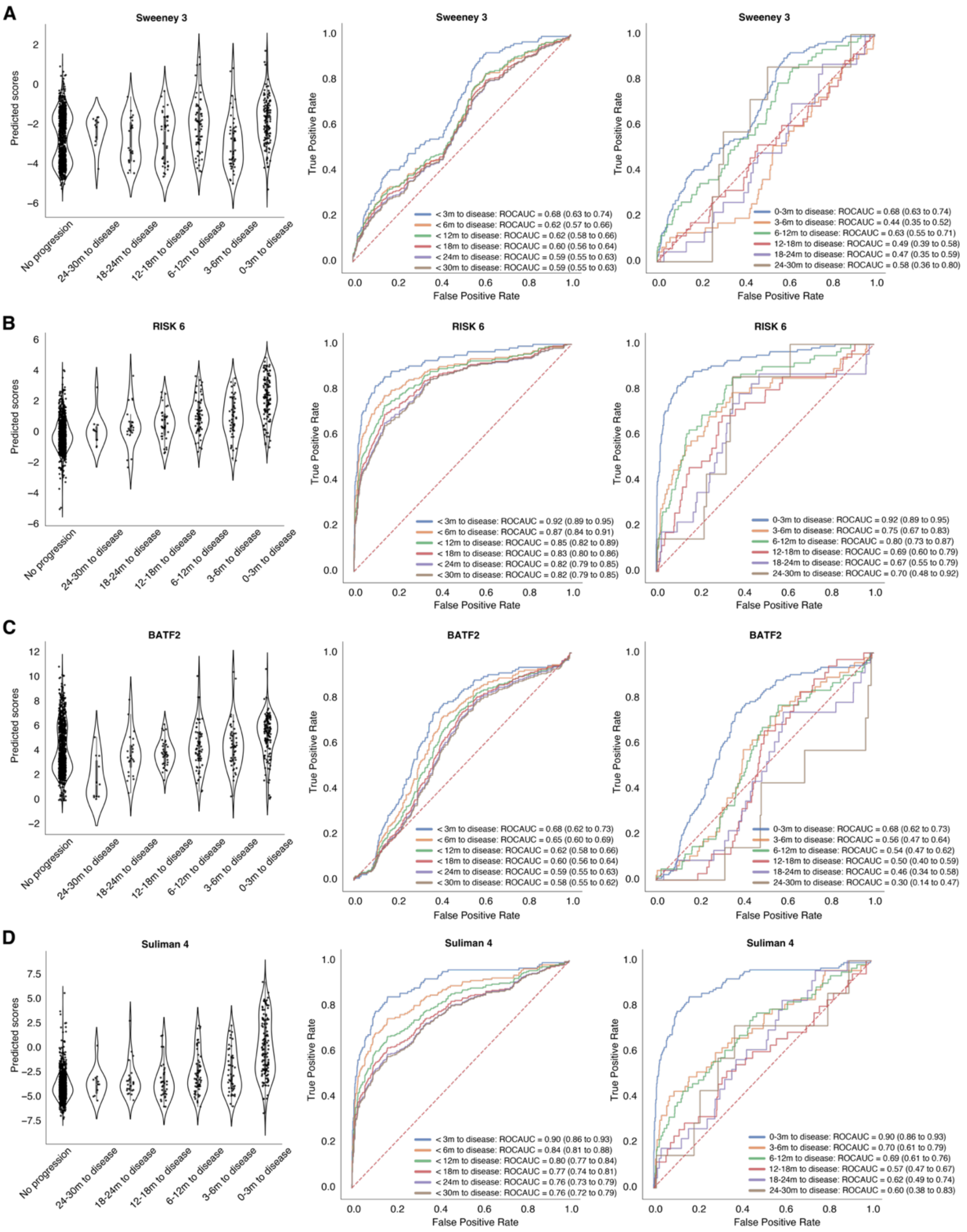

S9 Fig. Prognostic performance of the 4 published models (*3*, *5*, *18*, *27*)**.** (**A-D**) for incipient TB using the pooled longitudinal validation dataset (6 TB progression studies). The distributions of TB scores, stratified by categorical interval to disease, are shown in a violin plot (datapoints *n* = 1281) (left panels). ROC curves depict prognostic performance for incipient TB, stratified by time intervals to disease (< 3, < 6, <12, <18, < 24, < 30 months) (middle panel) and mutually exclusive time intervals to disease (0-3, 3-6, 6-12, 12-18, 18-24, 24-30 months) (right panel). AUC and 95% confidence intervals for each interval to disease are shown.
