# Supplementary material for: Gene signature discovery and systematic validation across diverse clinical cohorts for TB prognosis and response to treatment
Source: PLoS Comput Biol. 2023 Jul 20;19(7):e1010770. doi: 10.1371/journal.pcbi.1010770 (PMC10393163; doi:10.1371/journal.pcbi.1010770)
Supplement: S10 Fig — Cohort study metadata can be found in Tables 1 and S1 (datapoints n = 6290). (A) The distributions of TB scores, stratified by different TB disease stages (HC, LTBI, and ATB), other lung disease (OLD) and viral infection (VI), are visualized in the violin plots. (B) ROC curves depict diagnostic performance of the models. AUCs and 95% confidence intervals for each comparison are also shown. Same analysis for full model shown in Fig 3E and 3F. (PDF) [file pcbi.1010770.s016.pdf]

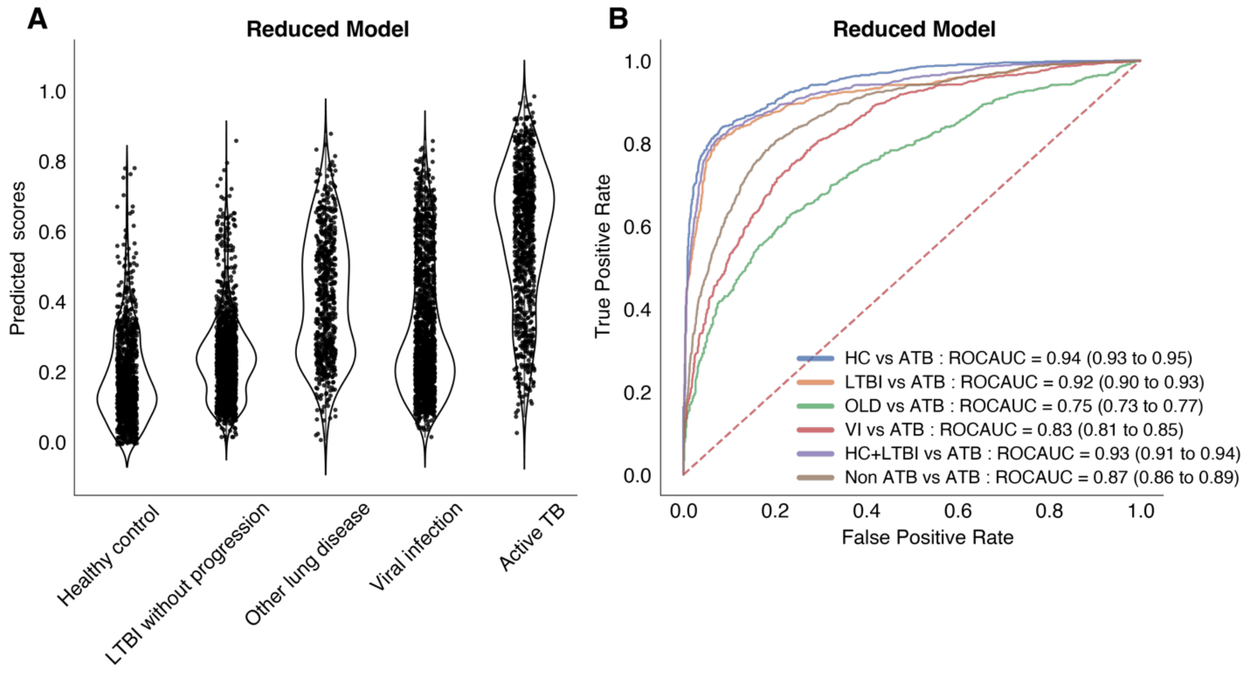

S10 Fig. Diagnostic performance of reduced model for active TB using a pooled dataset of all 57 collected cohort studies (**Table 1**, **S1 Table**) (datapoints n = 6290). (**A**) The distributions of TB scores, stratified by different TB disease stages (HC, LTBI, and ATB), other lung disease (OLD) and viral infection (VI), are visualized in the violin plots. (**B**) ROC curves depict diagnostic performance of the models. AUCs and 95% confidence intervals for each comparison are also shown. Same analysis for full model shown in **Fig 3E-F**.
