# Supplementary material for: Gene signature discovery and systematic validation across diverse clinical cohorts for TB prognosis and response to treatment
Source: PLoS Comput Biol. 2023 Jul 20;19(7):e1010770. doi: 10.1371/journal.pcbi.1010770 (PMC10393163; doi:10.1371/journal.pcbi.1010770)
Supplement: S12 Fig — The temporal dynamics of TB scores generated by the reduced model, stratified by the time of sputum culture conversion to negative (negativity at day 28, 56, 84 and 168, and no conversion at day 168 [failed]). The dashed line represents the TB scores of each patient responding to treatment over time, and the red line represents the median of the stratified group with 95% confidence interval shown in the shaded area. (PDF) [file pcbi.1010770.s018.pdf]

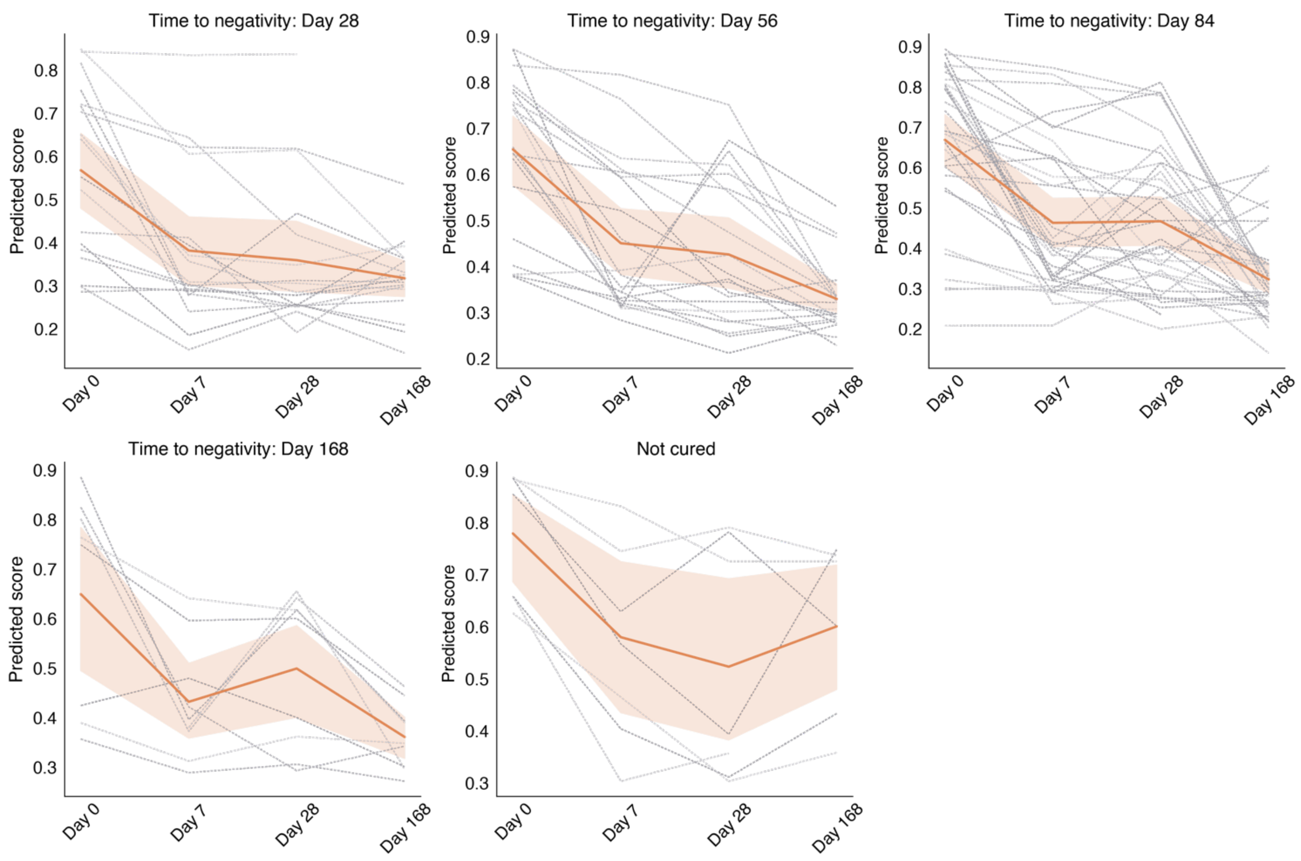

S12 Fig. The temporal dynamics of TB scores generated by the reduced model, stratified by the time of sputum culture conversion to negative (negativity at day 28, 56, 84 and 168, and no conversion at day 168 [failed]). The dashed line represents the TB scores of each patient responding to treatment over time, and the red line represents the median of the stratified group with 95% confidence interval shown in the shaded area.
