# Supplementary material for: Gene signature discovery and systematic validation across diverse clinical cohorts for TB prognosis and response to treatment
Source: PLoS Comput Biol. 2023 Jul 20;19(7):e1010770. doi: 10.1371/journal.pcbi.1010770 (PMC10393163; doi:10.1371/journal.pcbi.1010770)
Supplement: S13 Fig — ROC curves depicting predictive performance of the model for discrimination between cured patients with or without TB recurrence within 2 years after treatment completion are shown in different colors, stratified by different timepoints after treatment initiation. AUC and 95% confidence intervals for each interval to disease are also shown. (PDF) [file pcbi.1010770.s019.pdf]

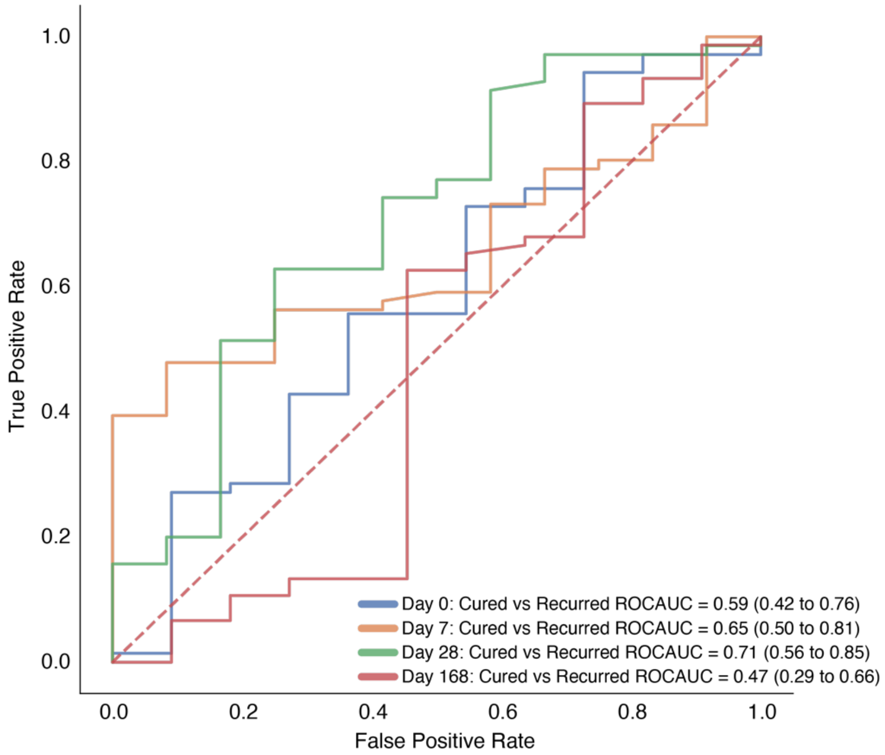

S13 Fig. Predictive performance of the reduced model in recurrent TB disease. ROC curves depicting predictive performance of the model for discrimination between cured patients with or without TB recurrence within 2 years after treatment completion are shown in different colors, stratified by different timepoints after treatment initiation. AUC and 95% confidence intervals for each interval to disease are also shown.
