# Supplementary material for: Gene signature discovery and systematic validation across diverse clinical cohorts for TB prognosis and response to treatment
Source: PLoS Comput Biol. 2023 Jul 20;19(7):e1010770. doi: 10.1371/journal.pcbi.1010770 (PMC10393163; doi:10.1371/journal.pcbi.1010770)
Supplement: S14 Fig — ROC curves, stratified by different timepoints after treatment initiation, depict predictive performance of the models for discrimination between patients with bacteriological cure and those with treatment failure at EOT (A) and cured patient with or without TB recurrence within 2 years after treatment completion (B). AUC and 95% confidence intervals for each interval to disease are also shown. (PDF) [file pcbi.1010770.s020.pdf]

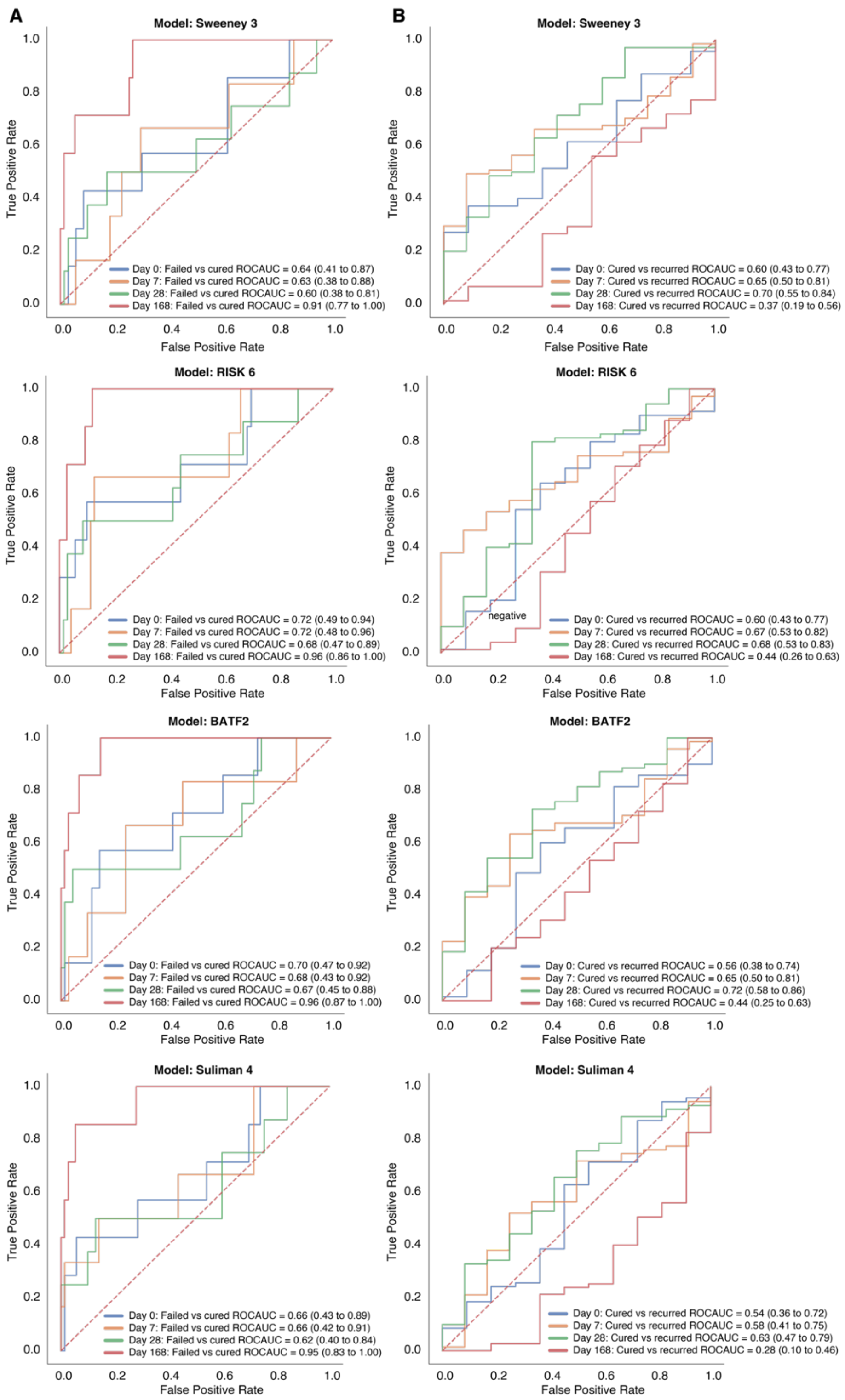

S14 Fig. Predictive performance of the 4 published models. ROC curves, stratified by different timepoints after treatment initiation, depict predictive performance of the models for discrimination between patients with bacteriological cure and those with treatment failure at EOT (**A**) and cured patient with or without TB recurrence within 2 years after treatment completion (**B**). AUC and 95% confidence intervals for each interval to disease are also shown.
