# Supplementary material for: Gene signature discovery and systematic validation across diverse clinical cohorts for TB prognosis and response to treatment
Source: PLoS Comput Biol. 2023 Jul 20;19(7):e1010770. doi: 10.1371/journal.pcbi.1010770 (PMC10393163; doi:10.1371/journal.pcbi.1010770)
Supplement: S15 Fig — The Spearman correlations between TB scores and MGIT culture time to positivity (A), Xpert Ct values (B), total glycolytic ratio activity (TGRA) at three time points after treatment initiation (Day 0, Day 28 and Day168) (C-E) are displayed. The scores at baseline stratified by radiologically persistent or cleared lung inflammation at EOT are shown in the violin plot (F). (PDF) [file pcbi.1010770.s021.pdf]

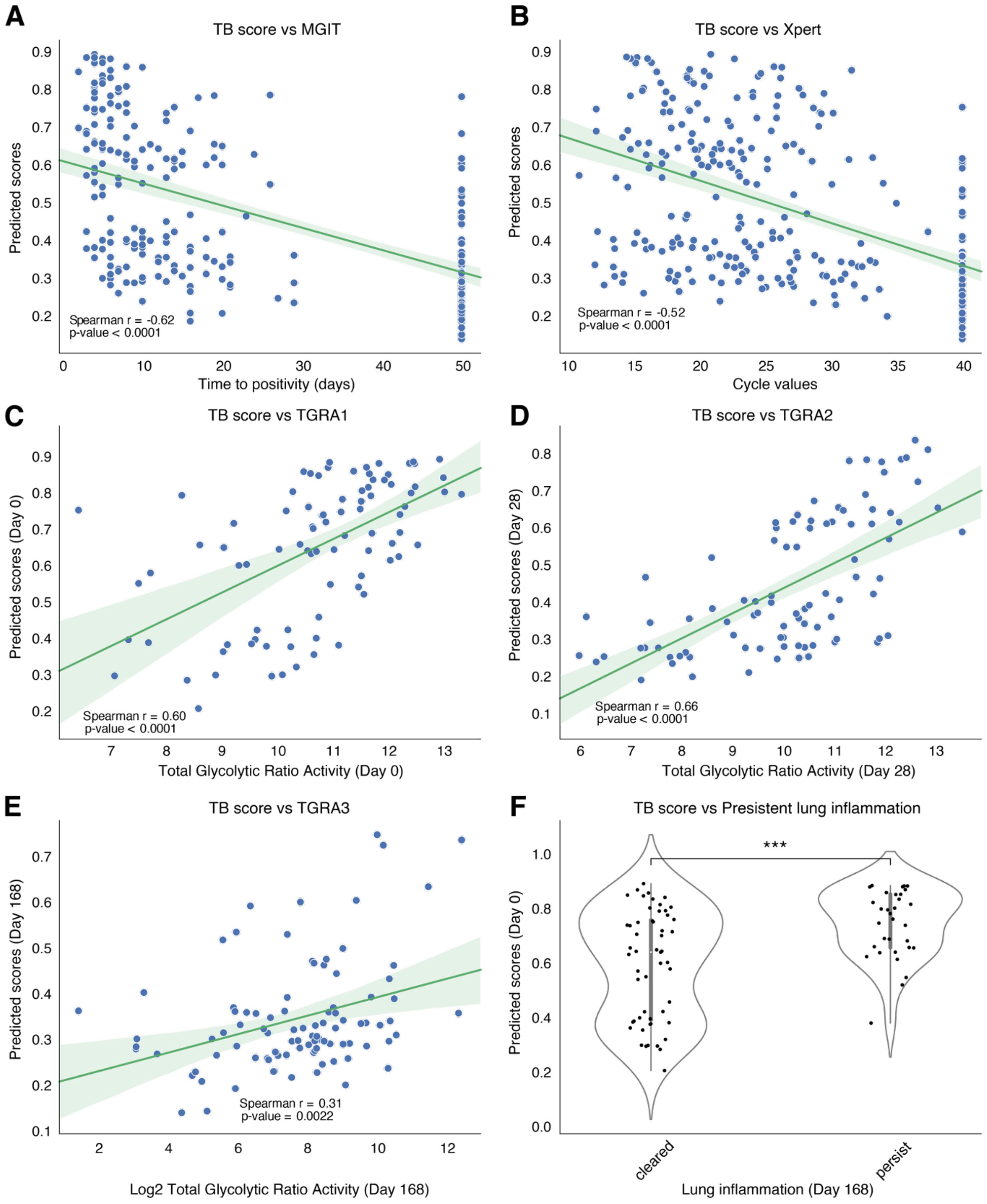

S15 Fig. The relationship between TB scores generated by the reduced model and *in vivo* pulmonary inflammation. The Spearman correlations between TB scores and MGIT culture time to positivity (**A**), Xpert Ct values (**B**), total glycolytic ratio activity (TGRA) at three time points after treatment initiation (Day 0, Day 28 and Day168) (**C-E**) are displayed. The scores at baseline stratified by radiologically persistent or cleared lung inflammation at EOT are shown in the violin plot (**F**).
