# Supplementary material for: Gene signature discovery and systematic validation across diverse clinical cohorts for TB prognosis and response to treatment
Source: PLoS Comput Biol. 2023 Jul 20;19(7):e1010770. doi: 10.1371/journal.pcbi.1010770 (PMC10393163; doi:10.1371/journal.pcbi.1010770)
Supplement: Attachment — Submitted filename: Response to Reviewers.docx [file pcbi.1010770.s022.docx]

**Reviewer #1**

Vargas and colleagues present a network-based meta-analysis approach using whole blood transcriptome data from multiple cohorts to identify a shared gene signature of active TB (ATB) infection. This work's major contribution is identifying a set of genes that can be assembled as a machine-learned signature that discriminates active ATB infection from healthy controls, latent TB, and other diseases across highly heterogeneous cohorts. Until now, no signature has been accurate across studies – this work, therefore, represents a major step forward for the field. They then train two ML models on the pairwise expression ratios of a subset of the identified genes to predict a TB score representing the spectrum of TB disease and treatment response.

**Major Suggestions**
1.1 It is unclear why a complex network approach to selecting the gene sets is preferred over simply choosing commonly differentially expressed genes and pathways among the heterogeneous studies. Given that the selected genes did not distinguish ATB from OD (or Tx), I wonder whether a set of common over-represented genes may perform better in the ML models.

Response 1.1: We recognize that many existing approaches can be used to select the represented genes fed into the ML model. Given the heterogeneity present in TB cohort studies due to varying demographics, disease comorbidities, and various TB disease states, we believe that only looking for differentially expressed genes across studies would most likely lead to little overlap in the genes between cohorts and disease states, or a few over-represented genes with no functional relationship. We show indirect evidence of this in **Fig. S4** in which we compare the existing gene signatures from previous reports that were derived by selecting genes that were differentially expressed within the studies’ respective cohorts. We observe no genes that are consistently identified across the studies.

We note that this limited commonality among the signatures could be driven by heterogeneity in the cohorts or a difference in computational approaches used to derive the signatures. This is the major reason that we decided to develop this network-based meta-analysis approach, to identify a common gene signature. The method contains two components. First, we identify genes that commonly show differential expression across the studies as well as different disease conditions (ATB vs HC, ATB vs LTBI, ATB vs OLD and ATB vs Tx) which is similar to other reports. Additionally, we considered genes in which the expressions are highly co-varied across the studies. By building a gene covariation network, the genes that are function-related and expressed consistently across the studies tend to be localized in the center of the network. As a result, we demonstrate that the 45-genes commonly appeared in all 27 studies and the gene set was enriched for genes actively involved in interferon activation responding to infection. Therefore, we believe this provides a complementary and unbiased approach to identify commonly differentially expressed, and functionally related gene sets, without using pre-defined pathway information.

Finally, we observe that there are only a few genes, such as HP and AEACAM1, that are expressed consistently in ATB vs HC, ATB vs LTBI, and ATB vs Tx but not in the ATB vs OLD comparison. However, not every gene in the 45 gene set was selected in the final ML models (full and reduced). During ML model training, the model went through two additional steps of feature selection, including univariate and multivariate feature selection (the details can be found in the **Materials and Methods/***Feature generation and selection*), after we generated an expression ratio between every pair of the 45-genes as the features that were fed into the ML model. As a result, the full model still considered HP but not AEACAM1, in which HP was in a paired ratio with C1QB as one of the features (**Supplementary Table 4**), whereas the reduced model did not take either of the genes as an input (**Supplementary Table 4**).

1.2 Neither the Catalysis nor the Leicester treatment response cohorts used for validation had HIV-positive patients. The authors should either validate their models’ predictions for this patient population or avoid stating that it could be reliably used for treatment outcomes in countries with high HIV burdens.

Response 1.2: We have rephrased this sentence in the **Discussion** section to avoid making this claim. In the previous MS draft this read -

“This provides additional evidence that our gene signature can be applied for TB risk estimation in high HIV burden countries.”

It now is written as follows -

“This demonstrates the potential for applying gene signatures for TB risk estimation in high HIV burden countries, however additional validation studies acquiring datasets with HIV+ individuals will be required to investigate this further.”

1.3 It is not clear how genes were determined in the reduced model. The motivation for creating the reduced model is briefly discussed near the end of the paper, but the derivation is mostly ignored. Based on the network in Supplementary Figure 4, many of the genes that are chosen in this reduced model are not very heavily connected, which was made out to seem like a critical criterion in the 45-gene model.

Response 1.3: We have modified some text into the **Results/Two novel machine-learning (ML) models predict active tuberculosis** section to motivate and make the derivation of the reduced model (from the full model) clearer to the reader -

“We generated a final RF model trained using the full discovery dataset; we refer to this model as the full model, its parameters are listed in **Supplementary Table 4.**

Next, blood transcriptomic signatures hold great promise for development of screening and triage tests but they impose a limit on the number of genes they can probe (*30, 32, 38*), we sought to construct a model with fewer features but without costing predictive performance. We further adapted a stability selection approach to perform a sensitivity analysis and down-selected the most robust features while preserving predictive performance (**Supplementary Fig. 6**). Using a forward stepwise selection within a 5-fold nested CV framework, we evaluated the RF algorithm by adding one feature at a time starting from the top ranked features. This final model with 12 features (paired genes) was determined with slightly reduced performance (average outer cross-validation AUROC = 0.85) (**Supplementary Fig. 7**) and refer to this model as the reduced model.

As a result, we generated two models, labeled as the full and reduced model (**Supplementary Table 4**)”

Full details on the derivations of the full and reduced models can be found the **Materials and Methods/Establishing optimized predictive models** where the sub-section *Feature generation and selection* and **Supplementary Fig. 6-7** contain the details to how features from the full-model were selected for inclusion in the reduced model.

With regards to the observation that many of the genes comprising the reduced model in **Fig. 2F** (previously **Supplementary Fig. 4**) not being connected, 12/18 = 67% of the genes in the reduced model are connected to the network, while 33/45 ~ 73% in the full model are connected to the network. Both models contain a similar proportion of genes connected to the network in **Fig. 2F**. Moreover, the emphasis of genes being heavily connected (being in the center of the network) principally applies to the gene selection criteria from the different comparison networks (ATB v HC, ATB v LTBI, ATB v OLD, and ATB v Tx) based on co-variation of gene expression across the cohorts (**Fig. 2A-D**)**.** In contrast, the network in Fig. 2F was the protein-protein association network using the STRING database and was intended only to illustrate the biological connection between the 45-gene set once it was selected and has no impact on downstream model performance for either the full or reduced model.

1.4 The writing is difficult to follow – as a result, there are details and nuances I cannot understand or review. In some areas, insufficient information is given (how were the networks assembled? How were the genes determined in the reduced model?). And in other areas, it was difficult to understand what was done and why (the paragraph about PPV and NPV). Paragraphs are long and dense and would benefit from section headings based on conclusions, not processes.

Response 1.4: We thank the reviewer for this feedback. We have revised the manuscript throughout to provide more details and to make the writing and logic clearer where appropriate. We have broken up several long paragraphs into smaller, more concise pieces, and have altered section headings in the **Results** to reflect the conclusions made in each respective section. A manuscript with these tracked changes has been uploaded for review.

1.5 Is the reduced gene set predicted to perform better as the number of cohorts is increased?

Response 1.5: Building a predictive model for TB prognosis and treatment monitoring has two major challenges. One is TB heterogenicity as we emphasized it in the **Introduction**. The other is that TB progression is dynamic spectrum from Mtb infection to subclinical/incipient disease, to non-severe and severe disease, instead of distinct disease states (LTBI or ATB). Given the public datasets we were able to collect, those datasets didn’t cover all the conditions such as different demographics, disease comorbidities, or disease states in the spectrum of TB disease. In theory, increasing the number of cohorts with regards to making the dataset more heterogenous to train the ML model’s hyperparameters will make the model more generalizable and potentially increases model performance. However, this is based on the assumption that there are indeed a few genes as ground truth that can be used to differentiate TB progression from all other clinical backgrounds or other diseases.

**Minor Suggestions**
1.6 Page 8, paragraphs 1 and 2: In paragraph 1, the authors state they utilize cohorts containing different clinical/genetic/technical covariates to “identify a set of common genes that robustly distinguish ATB from LTBI, HC, OD and ATB treatment.” The distinction between ATB and ATB treatment is made confusing by the use of “undergoing TB treatment (Tx)” to refer to the same group in paragraph 2 and in the rest of the paper. This should be made consistent throughout the manuscript.

Response 1.6: We thank the reviewer for pointing out this discrepancy. We have modified the first mention of Tx in the last paragraph of the **Introduction** section as follows: before it read -

“we first developed a network-based meta-analysis that utilized the collected cohorts containing different clinical/genetic/technical covariates to identify a set of common genes that robustly distinguish ATB from LTBI, HC, OLD and ATB treatment (Tx) across the cohorts”

Now it reads -

“we first developed a network-based meta-analysis that utilized the collected cohorts containing different clinical/genetic/technical covariates to identify a set of common genes that robustly distinguish ATB from LTBI, HC, OLD and undergoing TB treatment (Tx) across the cohorts”

To maintain consistency for the rest when referring to Tx throughout the manuscript.

1.7 Page 15, paragraph 2: The authors state – “Taken together, the results highlight that model only considering the minimized gene set […] may not be sufficient to deal with cohort heterogeneity.” In this sentence, “model” should be changed to “models” or “a model”.

Response 1.7: We have corrected this in the text, and the corresponding sentence has been changed to “Taken together, the results highlight that models only considering the minimized gene set […] may not be sufficient to deal with cohort heterogeneity.”

1.8 Figure 2, panel D: the ATB treatment group is abbreviated as “Tret”. Elsewhere in the paper, the same group is referred to as “ATB treatment” and “Tx”. This should be made consistent across the manuscript.

Response 1.8: We have changed the labels in Figure 2, panels K & O (previously D & H) from “ATB v Tret” to “ATB v Tx”.

1.9 The text states that “the full model was able to statistically separate the subjects’ scores between clinically defined subgroups and healthy controls as well as in-between clinically defined subgroups”, however, Figure 3B shows no significance in difference between the subclinical TB and clinical TB groups.

Response 1.9: We have added the following highlighted text into this sentence to clarify that there was no significant difference between subclinical and clinical TB groups -

“The full model was able to statistically separate the subjects’ scores between clinically defined subgroups and healthy controls as well as in-between clinically defined subgroups (with the exception of subclinical TB & clinical TB) (**Fig.** **3B**)”

1.10 The figure text is very small, making it difficult to read. I was unable to review the figures in printed format.

Response 1.10: We have increased the size of the font in all of the main and supplementary figures where possible. In some case, we were limited in the amount we could increase the font size by due to space constraints for that figure.

1.11 The figures should use color more deliberately. Within the same figure, red means no disease (Fig 6A) or severe disease (Fig 6B). It is not clear that color is needed at all in the violin plots, because they are labeled along the x-axis.

Response 1.11: We thank the reviewer for bringing this to our attention and agree that removing unnecessary color from figures aids in the readability of the manuscript. We have removed the use of color in all violin plots in the main and supplementary figures where appropriate (where the plots are labeled across the x-axis). We have made this change to Figures 3, 4, 5, 6, and Supplementary Figures 8, 9, 10, 11, 15.

1.12 Consider using fewer abbreviations. Some are nonstandard (OD reads as optical density to experimentalists!) and others (PPV and NPV) are not defined in the main text or in the first instance.

Response 1.12: We thank the reviewer for bringing to our attention the confusion that could stem using that using the abbreviation “OD”. We have changed this abbreviation for “other lung disease” to “OLD” instead, and have made the change throughout the main text & figures, and supplementary text & figures. Additionally, we have now defined PPV (positive predictive value) and NPV (negative predictive value) at first mention within the text in line 227.

1.13 Some language should be made more formal, for example “didn’t” appears several times.

Response 1.13: In part of our effort to make the manuscript more coherent in response to comment R1.4, we have removed all contractions from the text and have improved the grammar throughout to make the language more formal. A manuscript with tracked changes is attached for review.

1.14 Please consider adding line numbers – it makes it easier to comment on specific areas.

Response 1.14: We have added line numbers to the main manuscript file to aid in the review process.

1.15 Consider consolidating some of the supp into intuitive figures or tables for the main text. As written, a reader who does not download the supp materials (which is most readers) will have to believe and not see how the models were built and features selected.

Response 1.15: We thank the reviewer for the suggestion of moving some of the important information from the supplement to the main text to aid the reader in comprehending the results. To this end, we have incorporated what was previously **Supplementary Figure 3** (the distribution of weighted degrees across the different networks and the corresponding probability density functions), and **Supplementary Figure 4** (the PPI network from the 45-gene set) into **Main Figure 2A-F**. We believe this will help the reader better understand how the 45 genes were selected from the four ATB v HC, ATB v LTBI, ATB v OLD, ATB v Tx networks **(Fig. 2A-E),** and highlight the biological relevance of the 45 genes selected **(Fig. 2F)**. To further aid the latter point, we have also incorporated an illustrated version of **Supplementary Table 2** as **Fig. 2G** displaying the pathways (and specific genes) enriched in the 45-gene set. We have moved the distributions of predicted TB scores from the full model for patients separated into groups ATB, VI, OLD, LTBI, HC from a supplementary figure into the main as **Fig. 3E** to complement the ROC curves generated from these data in **Fig. 3F**. Finally, we have incorporated a condensed version of **Supplementary Table 1** as **Table 1** (we refer the reviewer to Response 1.16 for full details).

1.16 A table showing how data from the different studies were used in training and testing (or both) would help the reader follow how to evaluate independent tests of the models.

Response 1.16: We thank the reviewer for the suggestion to include a table to show how different datasets were used in this study. As there were many studies and a lot of associated metadata to include for each study, we have attached this table as an excel spreadsheet and have included it as **Table S1**. The table includes the GEO or EMBL-EBI accession IDs for each dataset, the breakdown of the patient types for each sample for the TB cohorts (ATB, LTBI, OD, Tx, HC), virus types for the respiratory virus cohorts, and whether the dataset was used in constructing the network models and in ML training/testing, or whether the datasets were used in ML validation. Additionally, to aid the reader in evaluating the independent tests of the models without having to download the supplementary file, we have included a condensed version of this table as **Table 1**.

**Reviewer #2**

Vargas et al., compiled 57 transcriptomic datasets from whole blood of tuberculosis (TB) patients, healthy individuals, or other diseases and used a combination of network modeling and machine learning to generate a new gene signature with the improved ability to distinguish active TB infection from healthy and other viral respiratory diseases. The model possesses an AUROC of 0.85 (74.2% sensitivity and 78.3% specificity) which is essentially in accordance with the WHO target product profile for prediction of progression to TB. Overall, I find the study to be well performed and interesting, possessing clinical relevance for an important global problem. I believe this manuscript warrants publication in Plos Computational Biology. I have detailed a few minor comments below:

**Specific comments**

2.1 I would encourage the authors to create a figure panel to support this assertion “The closer to the center (the higher weighted degrees) the nodes are, the more consistently the genes respond to active TB among various studies.” Perhaps by correlating gene log2FCs and gene network centrality across datasets/conditions?

Response 2.1: We thank the reviewer for the suggestion to include a visual aid in demonstrating the correlation between gene log2FC measurements against gene network centrality measurements across datasets & conditions. In **Fig. 2H-K** we plot the averaged log2FC for each gene against the weighted degree (indicated as network centrality) of that gene for each network constructed (ATB v HC, ATB v LTBI, ATB v OLD, and ATB v Tx). These plots resembled volcano plots routinely made for differential gene expression in which outlier genes with large changes in log2FC have correspondingly small p-values (in this case large weighted degree). Although we emphasize the patterns of the 45 genes selected with high log2FCs and high weighted degrees in each condition, plotting the genes in each network that show small log2FCs demonstrates that these genes have correspondingly low weighted degrees across conditions.

2.2 Some genes, like HP, appears to have extremely variable log2FC across datasets as evidenced from the heatmaps in Figure 2. To me, this warrants its exclusion from the final gene signature. Should the authors also include a requirement for low inter-dataset variability using variance or standard deviation of expression across datasets? I imagine genes with high variability across datasets might not be as reliable for clinical use. I leave it up to the authors to decide whether this would improve their results but encourage them to explore this.

Response 2.2: We certainly recognize the potential concern of selecting the gene like HP and AEACAM1 for clinical use. Notably, we identified the 45 genes that were in the top 5% of central genes from the weighted degree distribution of the network, were central in at least two of four networks, and had average logFC ≥ 0.5 or ≤ -0.5 across the studies. We intended to choose the best predictors that agreed in at least two out of four conditions but didn’t consider low inter-dataset variability across all datasets to further down-select the gene set. The major reason for this was that during ML model training, the model went through two additional steps of feature selection, including univariate and multivariate feature selection (the detail can be found in the **Materials and Methods/***Feature generation and selection*). We reasoned that two-step feature selections that evaluate the dependency between the features and the outcome would take into account the gene variability while training the model. As results, the full model still considers HP but not AEACAM1, in which HP was in pair with C1QB as one of the features (**Supplementary Table 4**), whereas the reduced model does not take either gene into account (**Supplementary Table 4**).

2.3 I would encourage the authors to provide figure evidence for this statement: “In addition, we plotted differential expression of the 45 genes in heatmaps across all datasets (Fig. 2E-H) and observed that gene patterns of upregulation and downregulation were consistent across cohorts within each disease condition and between disease conditions.” For instance, a condition correlation matrix where gene expression values are averaged for each condition and then correlated across conditions, or similar. In summary, it would improve the rigor if the authors provided an additional analysis to prove expression to be similar across conditions, which goes beyond merely displaying the heatmaps.

Response 2.3: We thank the reviewer for the suggestion in incorporating this additional analysis that strengthens our claim regarding the consistent patterns of upregulation and downregulation across the datasets & conditions. We constructed the condition correlation matrix suggested by averaging the logFC expression values across datasets within each condition for the 45-gene set and computing correlations pairwise. We have included the following highlighted text in the **Results/A network-based meta-analysis approach identifies a biologically meaningful common gene signature to active tuberculosis** section and corresponding **Supplementary Fig. 3** -

“In addition, we plotted the differential expression of the 45 genes in heatmaps across all datasets (**Fig. 2L-O**) and observed that gene patterns of upregulation and downregulation were consistent across cohorts within each disease condition and between disease conditions. Genes upregulated in ATB vs. HC for example were also upregulated in ATB vs. OLD, demonstrating a consistent signal for this gene set across cohorts within and between disease conditions. To validate this pattern for the 45-gene set, we averaged the logFC values for each gene across datasets within each comparison (ATB vs HC, ATB vs LTBI, ATB vs OLD, and ATB vs Tx) and computed the correlation between these averaged expression patterns between each pair of comparisons (e.g. ATB vs HC & ATB vs LTBI) to construct a correlation matrix (**Supplementary Fig. 3**). We observed that these differential expression patterns correlated strongly across disease conditions with r ranging between 0.61-0.96 and all p-values ≤ 1e-05. However, a few genes (such as HP, AEACAM1) showed slightly inconsistent expression patterns in ATB vs. OLD.”

**Supplementary Figure 3**. Pearson r correlation coefficient calculated between mean log2(Fold Change) between each pair of comparisons (ATB v HC, ATB v LTBI, ATB v OLD, ATB v Tx) for the 45 gene set. Correlations were computed by first averaging the values column-wise (across datasets) for the data in each heatmap **Fig. 2L-O** to calculate at vector of length *45* for each comparison, then calculating the Pearson r correlation (as implemented in *scipy* python package) between each pair of vectors. Correlation values and corresponding p-values are reported in the correlation matrix below.

The correlation results showed consistency with our co-variation network analysis, where the fold changes are highly correlated between ATB vs LTBI and ATB vs HC (r = 0.96). We also observed the largest overlap (66 genes) of the top 5% genes selected from the networks (ATB vs LTBI and ATB vs HC) (**Fig. 2E**). In contrast, the fold changes are less correlated between ATB vs Tx and ATB vs OLD (r = 0.61) and we only observed 1 gene overlap between the corresponding networks (**Fig. 2E**).

2.4 I would encourage the authors to move or re-create a gene set enrichment analysis of the 45 genes in the main, perhaps in Figure 2. The authors could also doing this for the smaller ML refined gene signature. The authors could even consider a clustergram with terms along the rows and genes along the column, indicating how each gene maps to each of the top X significantly enriched terms, though I leave the presentation choice to the authors. Presenting this visually the main figures will help the readers better appreciate the biology revealed by the gene signature.

Response 2.4: We thank the reviewer for the suggestion of creating a visual aid (clustergram) to aid the reader in understanding the biological relevance of enriched pathways and corresponding genes from the 45-gene set. We created a clustergram from the information present in **Supplementary Table S2**, and incorporated in the main text as **Fig. 2G** (which has been pasted below for reviewer convenience).

**Figure 2.** **Network analysis results and gene signature.** (**G**) A visualization of **Supplementary Table 2**, showcasing which pathways (and corresponding genes) were enriched for our 45-gene set (red: gene is in pathway, blue: gene is not part of pathway).

2.5 For the start of the machine learning section, a visual flow chart would be helpful to understand how features were defined and refined, and an indication of the size of the resulting final gene signature. In addition, the final ML refined gene set could be depicted as a heatmap (as in Figure 2) again in Figure 3.

Response 2.5: We agree with the reviewer that visually showing which genes were included in the reduced model, and the corresponding logFC across datasets and conditions could aid in the interpretation of the results. To this end, instead of recreating the heatmaps in **Fig. 2**, which would display redundant information, we have bolded the genes that are included in the reduced model. We have also denoted which genes (3/45) were not included in either the full or reduced model with an asterisk. **Fig. 2L-O** has been copied below. Further, we have amended **Fig. 1** to include a visual flow chart that shows how the models were trained and how the features were defined and refined, including the number of genes in each final model. **Fig. 1** has been copied below.

**Figure 2.** **Network analysis results and gene signature.** (**L-O**) Heatmaps of log2(Fold Change) for each of the 45 candidate genes (rows) between disease conditions displayed for each cohort (columns) that were used in constructing each network. The corresponding GSE ID of each cohort was labeled on columns. If a cohort contains multiple clinically defined populations, a specific comparison is also highlighted on the column. Bolded gene names are genes that are also included in the reduced model, genes with an asterisk are not included in either the full or reduced model.

**Figure 1. Network analysis results and gene signature.**

2.6 The respiratory virus control is an essential piece of this analysis. Without it, patients could be misdiagnosed, especially since the signature is composed of many inflammation-related genes. If possible, authors should include respiratory virus control in Figure 3. In general, the authors should include this control for all analyses, when possible. This was a major concern of mine when I approached this work.

Response 2.6: We agree with the reviewer on the importance of including the respiratory virus infection data into the analysis to ensure the models can discriminate between active TB (ATB) and viral infection (VI). We have included the results of this analysis for the full model in the **Results/Gene signature models discriminate active TB from non-active TB** and in **Fig. 3E-F**, both copied below.

“The full model demonstrated score distributions able to distinguish between the groups and provided a strong discrimination between ATB vs. HC, LTBI or OLD (**Fig. 3E-F**). Additionally, the full model was able to discriminate viral infection (VI) from ATB (AUROC 0.93, 95% CI 0.91-0.94), the overall AUROC between ATB and non-ATB (HC, LTBI, OLD and VI) achieved 0.92 (95% CI 0.91-0.93).”

We also performed this analysis for the reduced model, and have included the results in **Results/Gene signature models discriminate active TB from non-active TB** and in **Supplementary Fig. 10**, both copied below.

“As expected, the reduced model using fewer features had reduced diagnostic performance but maintained an AUROC at 0.87 (95% CI 0.86-0.89) when separating ATB from non-ATB **(Supplementary Fig. 10**). Its reduced performance was primarily attributed to insufficient differentiation between ATB and OLD (AUROC 0.75, 95% CI 0.73-0.77) and between ATB and VI (AUROC 0.83, 95% CI 0.81-0.85) (**Supplementary Fig. 10**).”

Finally, we also include the respiratory viral infection data in a similar analysis when assessing the discriminatory power for four previously published models in **Results/Gene signature models discriminate active TB from non-active TB** and in **Supplementary Fig. 11**, both copied below.

“In contrast, the pooled TB scores generated by the published models exhibited multimodal or dispersed distributions in multiple groups (**Supplementary Fig. 11**). This resulted in less overall discrimination between ATB and non-ATB (Sweeney 3 AUROC 0.81, 95% CI 0.79-0.83; RISK 6 AUROC 0.75, 95% CI 0.73-0.77; BATF2 AUROC 0.70, 95% CI 0.68-0.72; Suliman 4 AUROC 0.79, 95% CI 0.75-0.82) (**Supplementary Fig. 11**).”

2.7 The approach to use network modeling to refine the gene set is an interesting one with general applicability to other datasets and biological areas. However, one could imagine going directly from all genes into the machine learning algorithm or one could imagine alternative methods (e.g. lasso regression) to refine the gene set. At a minimum, the authors should comment on why they felt network modeling would out-perform other approaches (in the Discussion). Ideally, the authors should attempt to prove that incorporating the network modeling, versus excluding it, either (1) improves the predictive power of their resulting gene signature, (2) enhances the feasibility of the analysis, or (3) both.

Response 2.7: As is common in Data Science, we agree with the reviewer that when applying machine-learning models to biological data, an appropriate feature selection is an effective strategy to reduce feature dimension and redundancy and can alleviate the risk of multicollinearity and model overfitting. We considered several mathematical approaches to perform the feature selection, including lasso regression as the reviewer has mentioned. However, these methods mathematically consider the dependency between individual genes and the outcome, and do not necessarily account for biological co-variation between the genes across the cohorts. Given that the datasets we collected for our analyses are from diverse TB cohorts with varying demographics, disease comorbidities, sample collection, and profiling technologies, we prioritized leveraging the power of cohort/population heterogeneity to build a generalizable predictive model. To this end, we developed the network modeling approach to capture not only the most differentiated genes relevant to the outcome, but also to incorporate the covariation between genes that elucidated functionally important biological processes that underline TB progression.

Additionally, when we constructed the predictive model, instead of just considering individual genes, we used paired gene ratios as input into the model. This allowed us to not only consider first-order interaction between the genes, but also accommodate various data scales generated by different profiling technologies. If we had used all of the genes for which we had mRNA expression data, for model building (~10,000 genes), it would have resulted in >50 million features (paired genes) to consider (our approach considered 990 from every pairwise comparison of 45 genes). ML model benchmarking and its hyperparameter optimization with repeated nested cross validation would have become far less feasible and would have highly increased the risk of multicollinearity and model overfitting (p >> n problems).

We have incorporated the following text into the first paragraph of the **Discussion** section.

“When applying machine-learning models to biological data, an appropriate feature selection is an effective strategy to reduce feature dimension and redundancy and can alleviate the risk of multicollinearity and model overfitting. We considered several mathematical approaches to perform the feature selection, including lasso regression. However, these methods mathematically consider the dependency between individual genes and the outcome, and do not necessarily account for biological co-variation between the genes across the cohorts. Given that the datasets we collected for our analyses are from diverse TB cohorts, we prioritized leveraging the power of cohort and population heterogeneity to build a generalizable predictive model. To this end, we developed the network modeling approach to capture not only the most differentiated genes relevant to the outcome, but also to incorporate the covariation between genes that elucidated functionally important biological processes that underline TB progression.”

2.8 Some aspect of the analysis comparing to previously published signatures (Sweeney 3 and RISK 6) could be featured in the main as this will probably be of high interest to scientists in the field.

Response 2.8: We agree with the reviewer that the results from the previously published signatures may be of interest to other scientists in the field. Due to space constraints in the Main Figures, and our desire to keep the results from all previously published signatures in the same place, we have decided to display the results figures from all of the previously published signatures (Sweeney 3, RISK 6, BATF2, Suliman 4) in the Supplemental Section in **Figures S9, S11, and S14**. However, the performance of these models on all our analyses are mentioned in the **Results** text (with references to the Supplemental Figures). Further, the results of the prognostic performance of these published models on our validation datasets (in addition to the full & reduced models)

can be found in **Table2** and **Table3**.

2.9 In general, figure panels could be smaller and more compact to eliminate white space and maximize the transfer of information from main figures. Many interesting aspects from the supplement could be included in the main, for example, if space was optimized.

Response 2.9: We have altered all of the figures in the main and supplement to try and eliminate any unnecessary white space and as a result have moved several supplementary figures and tables into the main text. We refer the reviewer to **Response 1.15 & Response 1.16** for full details on which figures/tables were moved from the supplementary material

to the main text.

2.10 The title could be improved to communicate the most important finding of the manuscript. For instance, it is unclear to me why the word “common” is included in the title and feel it dampens the enthusiasm for the work. As an example: "Forty-five gene transcriptomic signature identifies TB infection across diverse patient cohorts”. In the end, however, I defer to the authors as to their choice of title.

Response 2.10: We thank the reviewer for their input on the wording of the title and have changed it to better communicate our findings, previously -

“Common gene signature model discovery and systematic validation for TB prognosis and response to treatment”

now -

“Gene signature discovery and systematic validation across diverse clinical cohorts for TB prognosis and response to treatment”
